# Crystal structure of dihydrofolate reductase from the filarial nematode *W*. *bancrofti* in complex with NADPH and folate

**DOI:** 10.1371/journal.pntd.0011303

**Published:** 2023-04-27

**Authors:** Keith Lange, Kathleen M. Frey, Tyler Eck, Cheryl A. Janson, Ueli Gubler, Nina M. Goodey

**Affiliations:** 1 Department of Chemistry and Biochemistry, Montclair State University, Montclair, New Jersey, United States of America; 2 School of Pharmacy and Health Sciences, Fairleigh Dickinson University, Florham Park, New Jersey, United States of America; NIAID: National Institute of Allergy and Infectious Diseases, UNITED STATES

## Abstract

Lymphatic filariasis is a debilitating illness with an estimated 50 million cases as of 2018. The majority of cases are caused by the parasitic worm *W*. *bancrofti* and additional cases by the worms *B*. *malayi* and *B*. *timori*. Dihydrofolate reductase (DHFR) is an established target in the treatment of cancer, bacterial, and protozoal infections and may be a potential target for drugs targeting parasitic worm infections, including filariasis. Recent studies have shown that known antifolate compounds, including methotrexate, inhibit the activity of *W*. *bancrofti* DHFR (*Wb*DHFR). However, the absence of structural information for filarial DHFRs has limited the study of more in-depth structure-function relationships. We report the structure of *Wb*DHFR complexed with NADPH and folate using X-ray diffraction data measured to 2.47 Å resolution. The structure of *Wb*DHFR reveals the usual DHFR fold and is currently only the second nematode DHFR structure in the Protein Data Bank. The equilibrium dissociation constants for NADPH (90 ± 29 nM) and folate (23 ± 4 nM) were determined by equilibrium titrations. The interactions of known antifolates with *Wb*DHFR were analyzed using molecular docking programs and molecular dynamics simulations. Antifolates with a hydrophobic core and extended linker formed favorable interactions with *Wb*DHFR. These combined data should now facilitate the rational design of filarial DHFR inhibitors, which in turn can be used to determine whether DHFR is a viable drug target for filariasis and whether existing antifolates may be repurposed for its treatment.

## Author summary

Lymphatic filariasis is a disease commonly characterized by urogenital swelling, or scrotal hydrocele and lymphedema, which affects nearly 50 million people worldwide as of 2018. It is caused by parasitic worms transmitted through mosquitos. Dihydrofolate reductase (DHFR) is a ubiquitous enzyme involved in folate metabolism and provides building blocks for DNA synthesis. DHFR is an established pharmacological target in the treatment of cancer and infectious diseases through the use of DHFR specific inhibitors known as antifolates. Using recombinant DHFR from the parasitic worm *W*. *bancrofti* (*Wb*), we have recently shown that known antifolate compounds (i.e. methotrexate and others) potently inhibit this enzyme. However, the absence of structural information for *Wb*DHFR has limited the investigation of in-depth structure-function relationships. We now report the first crystal structure of *Wb*DHFR. Using *in silico* approaches, a series of known antifolates was docked into the active site of the enzyme to gain insights into their interactions with *Wb*DHFR. Such data will facilitate the rational design of inhibitors specific for filarial DHFRs. The availability of such inhibitors will be crucial to establish DHFR as a valid target for the treatment of filariasis and will also help determine whether existing antifolates may be repurposed for treatment.

## Introduction

Lymphatic filariasis, also known as elephantiasis, is a mosquito-borne helminth infection prevalent in Sub-Saharan Africa and Southeast Asia. The disease is caused by the parasitic worms *W*. *bancrofti (Wb)*, *B*. *malayi (Bm)*, and *B*. *timori* [1]. Approximately 500 million people annually receive medications such as ivermectin, diethylcarbamazine, and albendazole to treat and prevent lymphatic filariasis infection [2]. Symptoms of lymphatic filariasis include swelling of the arms and legs, hydrocele, and fibrosis resulting in long-term disabilities. A significant proportion of patients carry co-infections with more than one parasite [1,3–5]. Some of these co-infections include malaria and schistosomiasis, a disease caused by another parasitic worm, *S*. *mansoni* (*Sm*) [6, 7].

Dihydrofolate reductase (DHFR) is a ubiquitous enzyme that catalyzes the conversion of dihydrofolate (DHF) to tetrahydrofolate, a key step in synthesizing pyrimidines such as thymidylate [8–12]. As the pyrimidine thymidylate is required for DNA synthesis, DHFR is a well-known drug target in cancer, bacterial and protozoal infections, and inflammatory diseases [13–16].

Literature reports have discussed the potential of DHFR as a drug target for treating helminth infections including filariasis [17–20]. Sharma and coworkers demonstrated that three antifolate compounds reduce *Bm* microfilariae motility by over 99% [17–18]. Upon treatment with folate, the authors observed motility returning, suggesting that inhibition of folate metabolism was responsible for the loss of motility. Supporting this is the fact that folate is structurally similar to the substrate DHF with the exception that in folate N8 and C7 are oxidized (Fig A in S1 Text). Furthermore, recent studies have demonstrated *in vitro* inhibition of filarial DHFRs from *Wb* and *Bm* by antifolate compounds [21, 22]. For example, methotrexate showed K_I_ values of 0.7 ± 0.1 nM and <0.5 ± 0.3 nM against *Wb*DHFR and *Bm*DHFR, respectively. While methotrexate also inhibits the human DHFR (*Hs*DHFR), and therefore is not a selective antifilarial agent, these data nevertheless suggest that antifolates may represent treatment options for filariasis [22]. However, the absence of structural information for filarial DHFRs has made the investigation of structure-function relationships difficult. Serrão and coworkers recently reported the X-ray structure of *S*. *mansoni* DHFR (*Sm*DHFR) (PDB code: 3VCO), the only other structure currently available for a nematode DHFR [23, 24].

**Fig 1:**
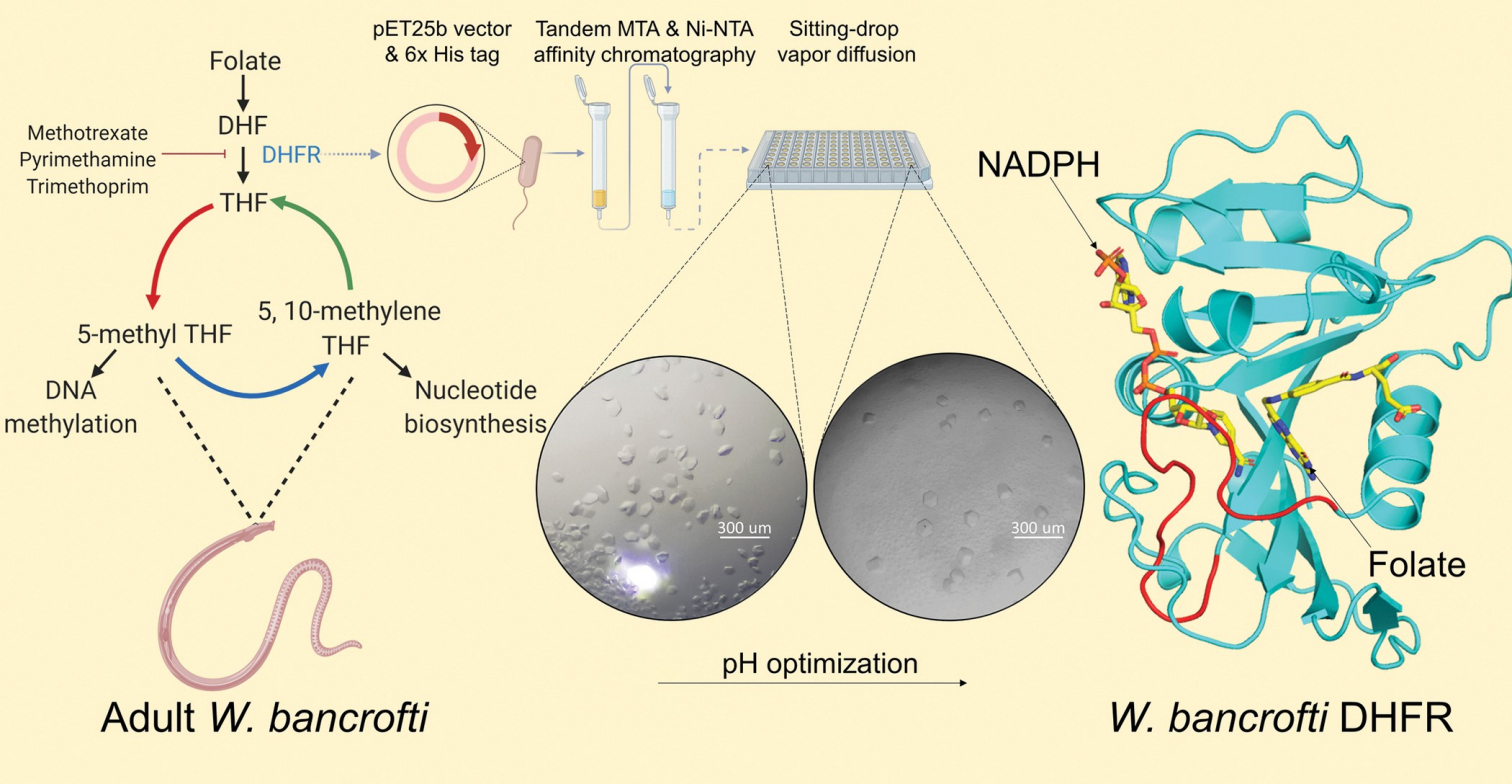
Overview of experimental design and results. The figure was created with BioRender.com.

Approximately 90% of filariasis cases are caused by *Wb* [25, 26]. Here we report the structure of *Wb*DHFR in complex with its cofactor NADPH and folate determined by X-ray crystallography (Fig 1). The resulting structure made it possible to dock a series of known antifolates into the active site, analyze interactions using molecular dynamics simulations, and gain insights into their interactions with *Wb*DHFR, as a possible guide for future drug design efforts.

## Methods and materials

### Expression and purification of *Wb*DHFR

The open reading frame of *Wb*DHFR including an engineered N-terminal His6-tag was expressed in the *E*. *coli* LOBSTR strain [27] in terrific broth (TB) with 100 μg/mL ampicillin at 37°C with shaking at 225 rpm. The starter culture was diluted 100-fold into 1 L of TB with 100 μg/mL ampicillin, grown to an OD_600_ of 0.8–1.5 and induced with 1 mM IPTG at 37°C. Cells were collected after 4 hours of incubation by centrifugation at 5,000 rpm for 30 minutes at 4°C using a JA-10 rotor in an Avanti J-26S XP centrifuge. The resulting 12–16 grams of wet pellet that originated from 1 L of culture were stored at -80°C until use.

The pellet from 1 L of culture as prepared above was resuspended in 100 mL of Mtx_Eq. buffer (8 mM Na_2_HPO_4_, 2 mM KH_2_PO_4_, 300 mM NaCl, 0.5 mM DTT, 10% glycerol, pH 7.0) and sonicated on ice for 15 minutes at 45% amplitude with 15 seconds on / 45 seconds off pulsing using the ½ inch probe on the Sonic Dismembrator FB505. The lysate was clarified by centrifugation at 17,000 rpm for 25 minutes at 4°C in a JA-17 rotor. The lysate was loaded onto a 5 mL methotrexate-agarose resin column (Sigma Aldrich) that had been equilibrated with 10 column volumes of Mtx_Eq. buffer. The column was washed with ~200 mL of Mtx_Eq. buffer and *Wb*DHFR was eluted with ~50 mL of 25 mM Tris, 300 mM NaCl, 5 mM folate, pH 8.6.

A nickel-nitriloacetic acid (Ni-NTA) column containing 10 mL of resin was equilibrated with 200 mL of 8 mM Na_2_HPO_4_, 2 mM KH_2_PO_4_, 300 mM NaCl, 10 mM imidazole at pH 7.4. The methotrexate-agarose column eluate was loaded directly onto the Ni-NTA column, and the column was washed with ~150 mL wash buffer (8 mM Na_2_HPO_4_, 2 mM KH_2_PO_4_, 300 mM NaCl, 35 mM imidazole pH 7.4). The *Wb*DHFR was eluted with 50 mL of 8 mM Na_2_HPO_4_, 2 mM KH_2_PO_4_, 300 mM NaCl, and 500 mM imidazole at pH 7.4. The Ni-NTA eluate containing *Wb*DHFR (~20 mL) was dialyzed against 4 L of 40 mM HEPES, 100 mM NaCl, pH 7.0 for one hour and against an additional 4 L of the same buffer overnight at 4°C. DHFR purity was assessed by SDS-PAGE (Fig B, part A in S1 Text). The concentration was determined at A_280_ using the molar extinction coefficient 25,440 M^-1^ cm^-1^ [22]. The protein was concentrated to 20 mg/mL using an Amicon Ultra-15 10,000 MWCO centrifugal filter. Purification of *Wb*DHFR yielded protein suitable for crystallization with a yield of 9–14 mg per liter of culture. The final *Wb*DHFR preparation was filtered, aliquoted, and stored at -80°C.

### Crystallization of *Wb*DHFR via sitting drop diffusion

*Wb*DHFR was buffer exchanged using a 10 kDa MWCO centrifugal filter into 20 mM HEPES, 25 mM NaCl, 5 mM DTT, and 5 mM NADPH at pH 7.0 and incubated on ice for one hour. Several crystallization screens from Hampton Research and Jena Biosciences were evaluated using sitting drop vapor diffusion in MRC 96-well plates. The lower reservoir contained 50 μL of precipitant solution and drops were made with one microliter protein solution (20 mg/mL) and one microliter precipitant solution. The Jena Biosciences JBScreen Classic HTS I condition containing 100 mM tri-sodium citrate pH 5.6, 25% w/v PEG 4000, and 200 mM ammonium sulfate at 4°C grew tiny crystals and this condition was then optimized. Increasing the pH from 5.6 to 6.6 in 0.2 pH unit increments resulted in larger, hexagonal crystals. The largest crystals grew to full size in about three weeks using a precipitant reservoir of 100 mM tri-sodium citrate pH 6.2, 25% w/v PEG 4000, and 200 mM ammonium sulfate. Crystals were cryo-protected in the precipitant solution containing 15% (v/v) glycerol. After transferring the crystals to the cryosolution, they were immersed in liquid nitrogen and stored in a cryo-Dewar until data collection.

### Data collection and structure determination

Diffraction data for all crystals were collected at the Brookhaven National Synchrotron Light Source II (NSLS-II) on beam line AMX (17-ID-1). A total of 1,800 0.2° images were collected spanning a phi range of 360°. All images were indexed, integrated, and scaled using XDS [28]. Molecular replacement (MR) using the previously solved structure of mouse DHFR (PDB code 2FZJ) with high homology (~40%) was used to obtain phases. The starting mouse model was modified using Sculptor in Phenix [29] and the *Wb*DHFR input sequence also included the 13 amino acid loop described in previous work [22]. The modified model was then used for MR using program Phaser [30]. Programs Phenix [29] and COOT [31] were used to build and refine the structure. Several iterations of model building and refinement continued until acceptable R-factors and geometric parameters were achieved. Bond lengths, angles, and clashes were assessed using MolProbity [32]. An unbiased omit *2 F*_*o*_*-F*_*c*_ map was generated using the “Iterative Omit Map Tool” in Phenix (Fig C in S1 Text) [29]. The final structure was analyzed in PyMOL and Maestro [33, 34].

### Molecular docking

The *Wb*DHFR ternary structure with NADPH and folate bound served as the starting model for docking studies. The programs AutoDock VINA [35] and Glide (Schrödinger) [36] were used to dock compounds in parallel. Three-dimensional structure files (.sdf) for all antifolates were obtained from the PubChem database. AutoDockTools 1.5.6 was used to generate the appropriate input.pdbqt files for the docked ligands and *Wb*DHFR. [37] The following center coordinates in Ångstroms were used for the docking grid in Autodock Vina [35]: x = 59.265, y = 17.612, z = -0.07. The models were analyzed in PyMOL [33]. To validate the docking methods, folate was first deleted from the ternary structure and then docked back into the active site of *Wb*DHFR, with a resulting docking energy score of -9.1 kcal/mol (Vina) and -9.0 kcal/mol (Glide). The predicted low docking energy scores correlate with the low K_D_ (23 nM) that was determined for folate experimentally. Docking results also showed folate binding in a similar conformation to that observed in the experimentally determined ternary *Wb*DHFR crystal structure, indicating that the docking approach was accurate. The antifolates methotrexate, pyrimethamine, trimethoprim, aminopterin, and trimetrexate were analyzed using molecular docking programs. The resulting docking models were used to predict contacts involved in drug-target interactions.

For comparison of docking scores obtained by another program, the same antifolates were docked into the *Wb*DHFR crystal structure using the molecular docking program Glide [36]. The *Wb*DHFR crystal structure was prepared using Protein Preparation in Maestro and the antifolates were prepared using Ligprep [34]. The docking grid was generated by specifying the 12 Å radius around folate in the *Wb*DHFR active site; the docking grid coordinates in Ångstroms: X = 55.92, Y = 16.12, Z = -0.8. The antifolates were docked to the grid using Glide XP Precision Docking. Docking scores and poses for antifolates in the *Wb*DHFR active site were examined in PyMOL [33] and Maestro [34].

### Molecular dynamics simulations

The *Wb*DHFR crystal structure, *Hs*DHFR crystal structure (PDB code 2W3M), and the docking models for antifolates (methotrexate, aminopterin, trimetrexate, trimethoprim, pyrimethamine, and cycloguanil) were used for molecular dynamics (MD) simulations and analysis. Models were prepared by adding hydrogens, assigning charges, capping the termini, and deleting non-interacting water molecules using the Protein Preparation tool in Maestro [34]. The prepared models were energy minimized, then used to build the system for MD.

Systems for MD were prepared using System Builder in Desmond [38]. We predefined the solvent model as TIP4P. The system was neutralized by the addition of Na^+^ and Cl^-^ ions, simulating concentrations of 0.15 M. The force field system was Optimized Potentials for Liquid Simulations (OPLS) and OPLS was applied to each system. Water molecules and ions around the enzyme were built into the system within the calculated orthorhombic box volume for soluble proteins. The average calculated box volume was = 240,000 Å^3^. All models were relaxed before simulation. Desmond was used to conduct MD simulations under the following conditions: canonical ensemble (NVT); Berendsen thermostat (temperature = 300°K); and Berendsen barostat (pressure = 1.01325 bar); timescale = 10 nanoseconds (ns) [38]. We used 10 ns (10 picoseconds/interval) to allow for system equilibration confirmed by protein root mean square deviation (RMSD) plots. A total of 1000 snapshots (10 picoseconds/interval) were generated for each complex. Simulations were analyzed for equilibration and convergence by examining root mean square deviation (RMSD) versus simulation time plots. Trajectories from each simulation were analyzed using Simulation Interaction Analysis and Simulation Event Analysis programs in Desmond [38].

### Determination of K_D_ of NADPH and folate

The dissociation constants (K_D_) of the ligands NADPH and folate were determined by monitoring changes in tryptophan fluorescence upon increasing ligand concentrations. For dissociation constant determination, only the Ni-NTA affinity chromatography purification method (leaving out the methotrexate-agarose column step) was used to ensure *Wb*DHFR was not bound to ligands during affinity studies. NADPH or folate was added to 400 nM *Wb*DHFR at room temperature and fluorescence values were recorded using a Fluoromax-4 (Horiba Jobin Yvon) spectrofluorimeter (ex: 290 nm, em: 340 nm, with 5 mm and 10 mm slits, respectively). The tested ligand concentrations ranged from 0 to 10 μM for NADPH and 0 to 4 μM for folate. The titrations were performed by adding one microliter increments of ligand to a quartz cuvette that contained 1000 μL of 400 nM enzyme in 1X MTEN (50 mM MES, 25 mM Tris, 25 mM ethanolamine, 100 mM NaCl, 2 mM DTT) buffer at pH 6.0 and allowing equilibration for 10 seconds. The data were corrected for the inner filter effect using 200 nM L-tryptophan as described previously [39]. Fluorescence intensity values were graphed against ligand concentrations using Kaleidagraph and the data were fitted to the Morrison equation [40–42].

## Results and discussion

### Crystal optimization and structure determination

We here report the first crystal structure of DHFR from *Wb*, a parasitic nematode that causes lymphatic filariasis. The crystals obtained for data collection first appeared after five to eight days of incubation and continued to grow for three weeks (Fig B, part B in S1 Text). Crystals of *Wb*DHFR grew in the presence of NADPH in the crystallization buffer. One crystal diffracted to amplitudes extending to 2.47 Å using native wavelengths (1.0 Å). The diffraction pattern confirmed that the *Wb*DHFR crystals had a hexagonal lattice and space group P6_3_22. The data set was 99.69% complete with acceptable R-factors (Table 1). The Matthew’s coefficient predicted one molecule in the asymmetric unit (ASU) with ~ 75% solvent content. These parameters were used to solve the phases via molecular replacement with Phaser [30]. The structure was deposited in the Protein Data Bank as 8E4F. Crystallization trials including either methotrexate or pyrimethamine did not result in crystal growth. In fact, neither co-crystallization nor soaking pre-existing crystals with antifolates provided crystals of sufficient quality for diffraction studies.

**Table 1:** Data collection and refinement statistics.

	*Wb*DHFR
**PDB Code**	8E4F
**Wavelength** (Å)	1.00
**Resolution range** (Å)	28.99–2.472 (2.56–2.472)
**Space group**	P6_3_22
**Unit cell** (a = b = c = Å; α = β = γ = °)	a = 133.877 b = 133.877 c = 75.876 α = 90° β = 90° γ = 120°
**Total reflections**	29594 (2812)
**Unique reflections**	14808 (1417)
**Multiplicity**	2.0 (2.0)
**Completeness (%)**	99.69% (98.68%)
**Mean I/sigma(I)**	24.44 (4.94)
**Wilson B-factor (Å**^**2**^)	36.82
**R-merge**	0.01826 (0.1274)
**R-meas**	0.02583 (0.1802)
**R-pim**	0.01826 (0.1274)
**CC1/2**	1 (0.94)
CC*	1 (0.984)
**Reflections used in refinement**	14798 (1416)
**Reflections used for R-free**	741 (71)
**R-work**	0.2015 (0.2252)
**R-free**	0.2192 (0.2187)
**CC(work)**	0.941 (0.874)
**CC(free)**	0.934 (0.854)
**Number of non-hydrogen atoms (Total)**	1616
**Number of non-hydrogen atoms (Macromolecules)**	1441
**Number of non-hydrogen atoms (Ligands)**	93
**Number of non-hydrogen atoms (Solvent)**	82
**Protein residues**	182
**RMS (bonds =** Å)	0.006
**RMS (angles =** °)	0.90°
**Ramachandran favored (%)**	98.32%
**Ramachandran allowed (%)**	1.68%
**Ramachandran outliers (%)**	0.00%
**Rotamer outliers (%)**	5.84%
**Clashscore**	6.01
**Average B-factor (Å**^**2**^)	33.88
**Macromolecules (Å**^**2**^)	33.29
**Ligands (Å**^**2**^)	37.84
**Solvent (Å**^**2**^)	39.85

Note: Statistics for the highest-resolution shell are shown in parentheses.

### Analysis of the overall structure

**Fig 2:**
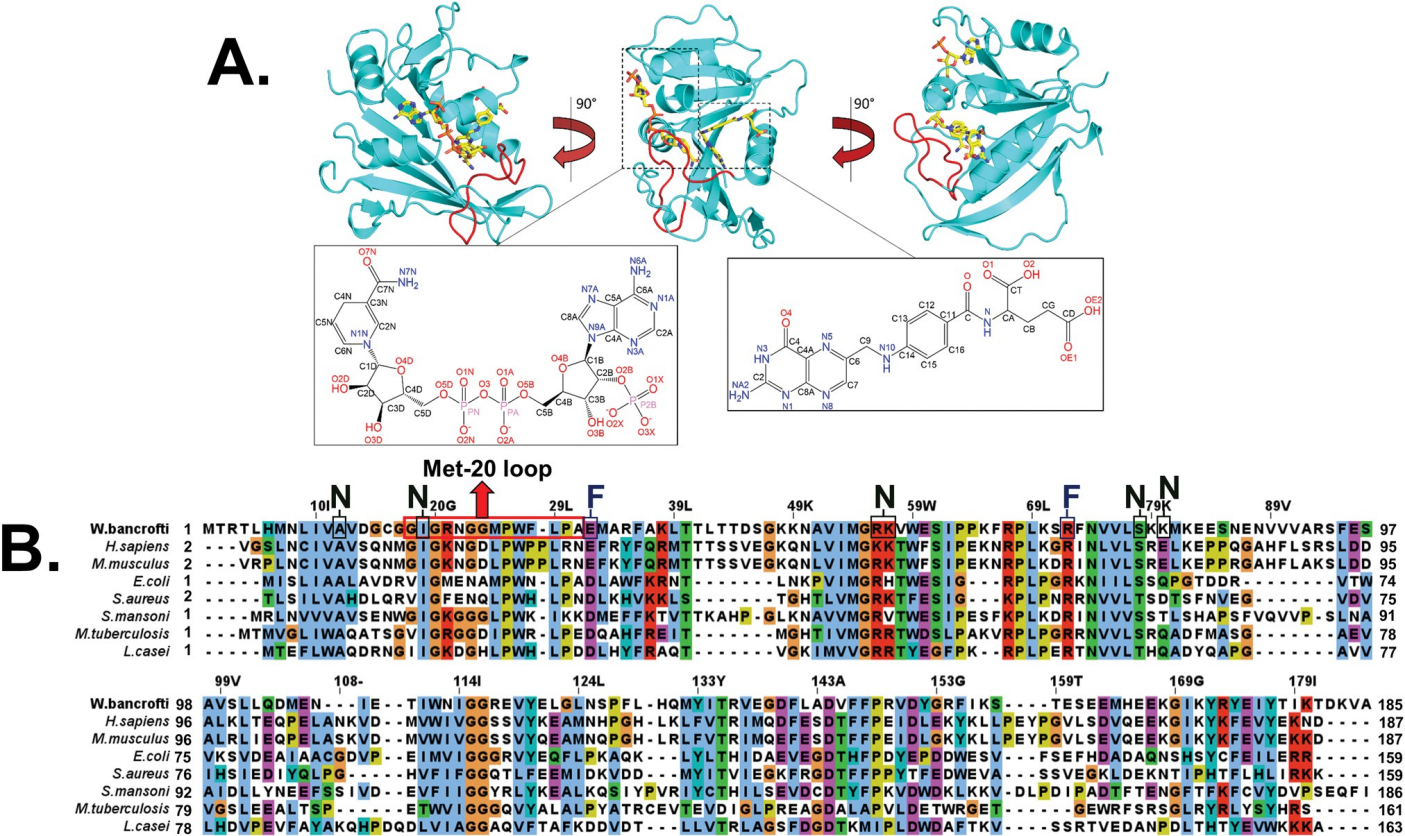
A ribbon diagram of *Wb*DHFR (cyan) shows the bound complex with folate and NADPH (yellow) with the atom labels of folate and NADPH. (A). An amino acid sequence alignment of eight prokaryotic and eukaryotic DHFRs is shown (B). Amino acids corresponding to the Met20 loop are highlighted in red in A and in a red rectangle in B. Sequences of DHFR orthologs were obtained through UniProt and aligned with the multiple sequence alignment tool “ClustalW” within Jalview. Amino acids with similar chemical characteristics were colored using the “ClustalX” color scheme. Amino acids observed to make hydrogen bonds with either folate or NADPH are labeled “F” and “N”, respectively. The first amino acid residues for *Hs*, *Mm* and *Sa* DHFR are denoted as #2 because these sequence entries are derived from proteins purified from the organisms rather than cloned cDNA.

The finalized crystal structure revealed a typical DHFR fold with four alpha helices and six parallel strands and one anti-parallel beta strand, similar to the human and bacterial orthologs of DHFR (Fig 2). One notable difference between the *Hs*DHFR and *Wb*DHFR orthologs is the absence of four amino acids near the C-terminus in *Wb*DHFR (#160–164 in the human enzyme) [43]. The crystal structure reveals a ternary complex with both NADPH and folate present in the active site. NADPH was intentionally co-crystallized with *Wb*DHFR while folate was used for the methotrexate-agarose column elution and remained protein-bound throughout the purification process. Omit electron density shows strong peaks at 1.0 sigma for folate in the active site (Fig C in S1 Text). The refined crystal structure of *Wb*DHFR also shows the presence of two SO_4_^2-^ ions bound to basic residues Arg-21, Arg-117, and Arg-149. These ions likely originated from the crystallization solution. According to our model, the donor NADPH-acceptor DHF distance in *Wb*DHFR was estimated to be 3.5 Å, (Fig D in S1 Text) similar to the hydride transfer distances in *E*. *coli* DHFR (3.6 Å) and *Hs*DHFR (3.0 Å) [44–45].

### Interactions with folate

**Fig 3:**
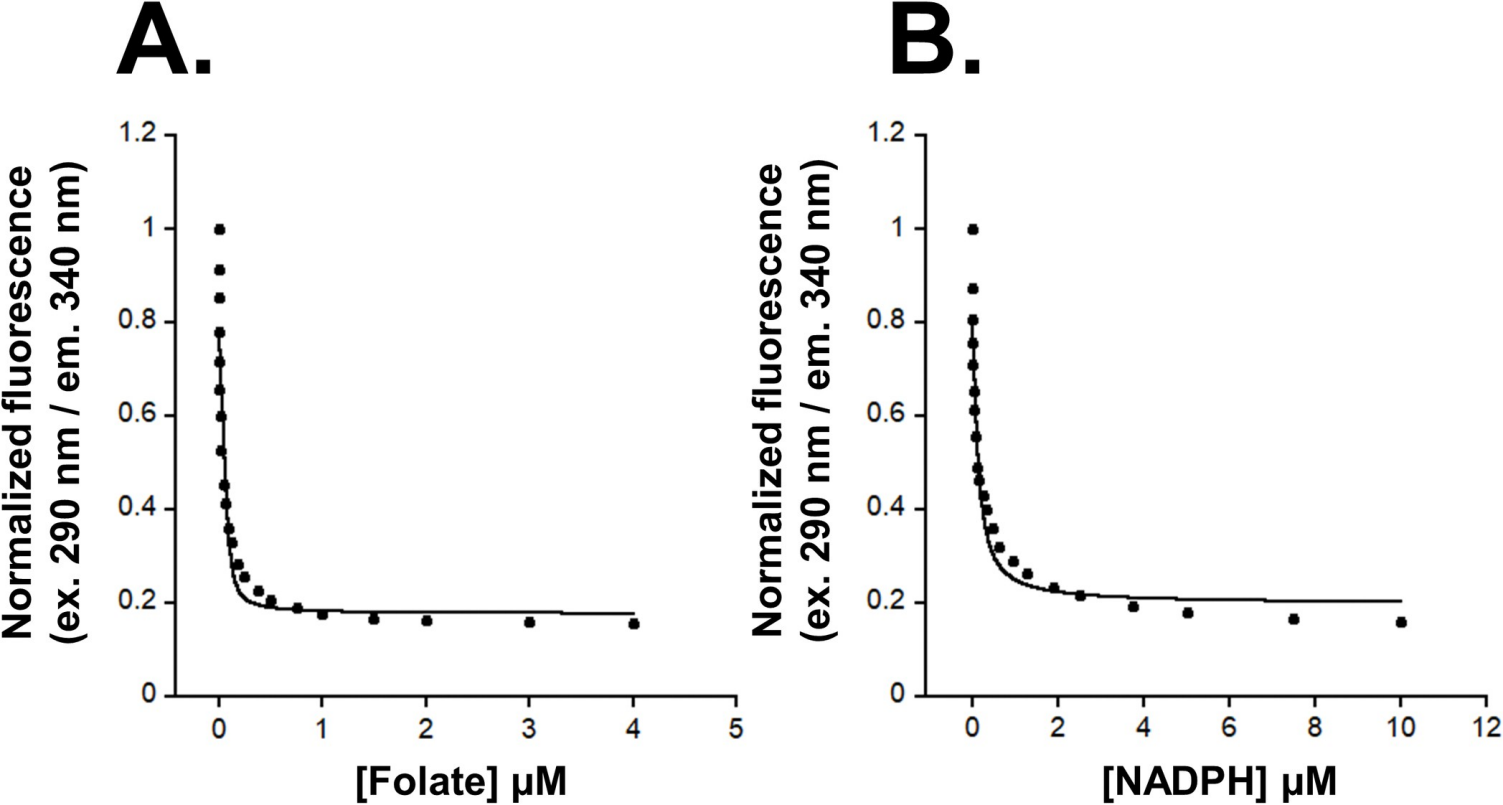
Determination of K_D_s of folate (A) and NADPH (B) with *Wb*DHFR using tryptophan fluorescence measurements. Approximately 400 nM of Ni-NTA purified *Wb*DHFR was titrated with folate (0–4 μM; A) and with NADPH (0–10 μM; B) in separate experiments. Fluorescence intensity (ex. 290 nm, em. 340 nm) of the sample was recorded using a Fluoromax-4 spectrofluorimeter. Three trials were normalized, averaged, and the data were fitted to the Morrison equation using Kaleidagraph [41].

**Fig 4:**
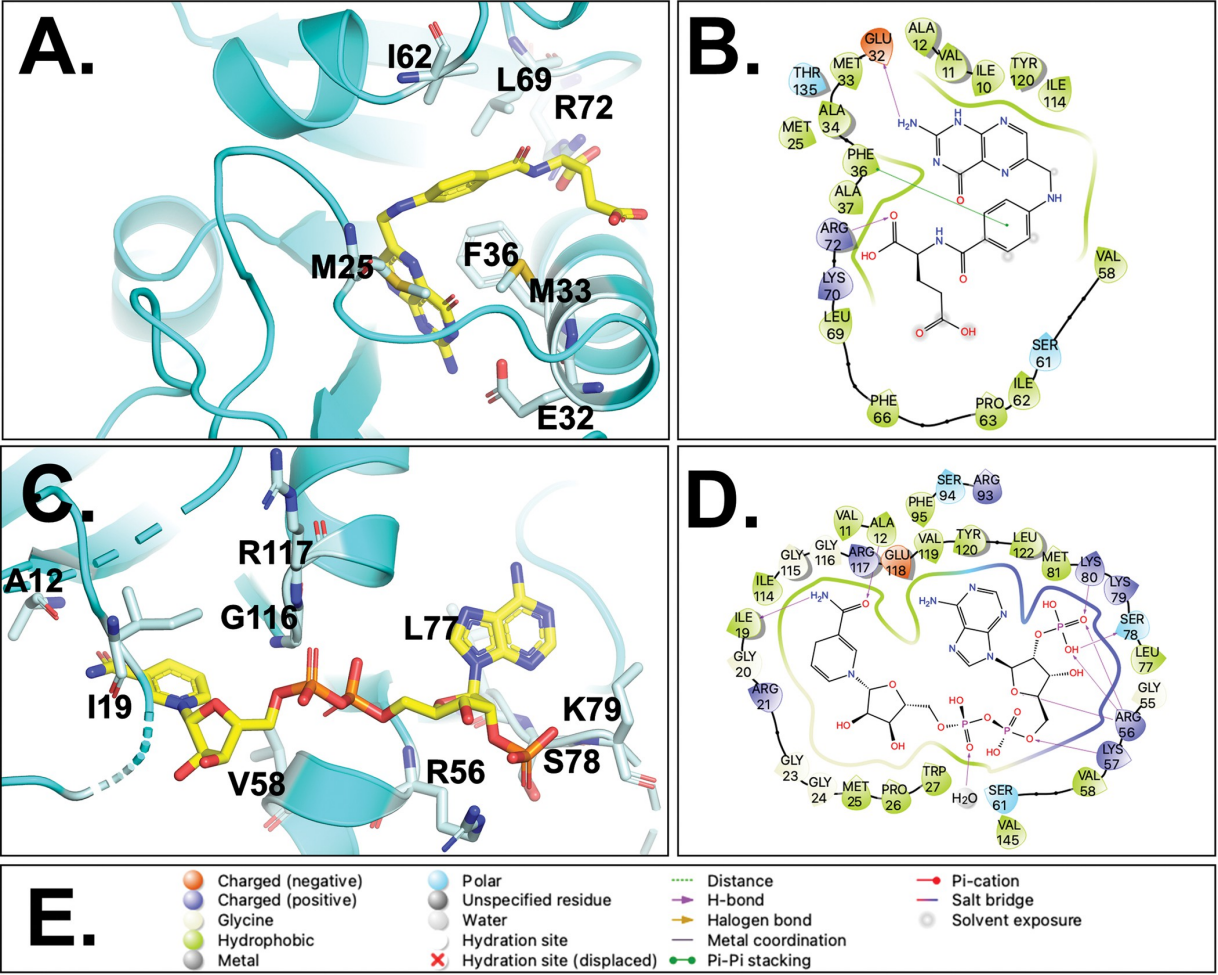
*Wb*DHFR (cyan) with amino acids involved in folate (A, B) and NADPH (C, D) binding are shown as light blue sticks. Three-dimensional representations (A and C) were generated in PyMOL while the two-dimensional images (B and D) were made in Maestro. Symbols used in B and D are described in E. The PDB entry 8E4F was used to generate these figures.

We determined the dissociation constant (K_D_) for folate to be 23 ± 4 nM, indicating tight binding affinity (Fig 3A). The low K_D_ for folate was measured in the absence of NADPH and we expect that the K_D_ would be even lower if NADPH was included in the folate K_D_ determination due to the reported positive cooperative binding of folate [46]. Using tryptophan fluorescence for K_D_ determination limits the experiment to only one ligand due to the need to correct for inner filter effects. Interactions observed for folate and residues of *Wb*DHFR include hydrogen bonds between the carboxylic acid of Glu-32 in *Wb*DHFR and the N3 and NA2 atoms of folate (Fig 4A and 4B; see atom labels in Fig A in S1 Text). The interaction distances between the carboxylate of Glu-32 and the N3/NA2 atoms are 2.9 Å and 2.6 Å, respectively (Fig D in S1 Text). The NH_2_ of Arg-72 hydrogen bonds with the O1 and O2 atoms of folate (Fig A in S1 Text), at distances of 3.0 Å and 2.7 Å, respectively (Fig D in S1 Text). Phe-36 is involved in π-stacking and makes van der Waals contacts with the para-benzoic acid group (PABA) of folate (Fig 4A and 4B). Phe-36 likely interacts with methotrexate in a similar manner in *Wb*DHFR; Phe-36 is usually mutated to serine or tryptophan in methotrexate-resistant DHFR enzymes [47–48].

### Interactions with NADPH

NADPH binds in the extended conformation identified in many DHFR structures [49]. Sequence differences in *Wb*DHFR compared with other DHFRs result in minor interaction changes between NADPH and the co-factor binding site. The K_D_ of NADPH and *Wb*DHFR (in the absence of folate) was determined using tryptophan fluorescence to be 90 ± 29 nM (Fig 3B). The K_D_ for NADPH binding to *Bm*DHFR, a filarial nematode ortholog with 96% sequence identity to *Wb*DHFR, was previously found to be 25 ± 24 nM [21]. These values are similar to the K_D_ (150 ± 20 nM) reported for the *E*. *coli* ortholog [50]. In DHFR homologs from *H*. *sapiens*, *E*. *coli*, *and C*. *albicans* (PDB codes 4KD7, 2ANO, 1AOE, respectively), the hydroxyl group and backbone NH of the highly conserved threonine, which corresponds to Val-58 in *Wb*DHFR, form hydrogen bonds with the pyrophosphate region of NADPH. In contrast, *Wb*DHFR contains a valine residue at position 58. The backbone NH of Val-58 forms a hydrogen bond with NADPH while the valine sidechain contacts folate via van der Waals interactions (Fig E in S1 Text).

In addition, *Hs*DHFR and *Mm*DHFR structures have a conserved arginine (Arg-77*)* that forms a sidechain π-cation interaction with the adenine ring. In *Wb*DHFR, this arginine corresponds to Lys-79. Electron density for the sidechain of Lys-79 was disordered and this side chain could not be modeled and therefore probably does not interact with NADPH.

The nicotinamide ring of NADPH (Fig 2A) is involved in π-stacking interactions with the pteridine of folate. This interaction is commonly found in ternary complexes of DHFR and highlights the cooperativity between DHF and NADPH binding sites. This stacking interaction also enhances the interactions of folate and residues Glu-32, Phe-36, and Arg-72 (Fig 4) by positioning the pteridine to maintain proper interaction distances.

### Comparison of *Wb*DHFR and *Sm*DHFR structures

To our knowledge, there is only one other nematode DHFR structure that is currently available in the PDB, the DHFR from *Sm*. In comparing *Wb*DHFR with *Sm*DHFR, we found that the structures are homologous (with an alignment RMSD of 1.324 Å) but differ in sequence; the amino acid sequence identity is only 32%. The alignment of the ternary *Wb*DHFR structure with the structure of the apoenzyme *Sm*DHFR revealed high structural similarity with the exception of the Met20 loop (Fig F in S1 Text). In *Sm*DHFR, the Met20 loop is disordered while in *Wb*DHFR, it exhibits a typical “closed” conformation (Fig F in S1 Text); these loop conformations are discussed in the article by Sawaya and Kraut [51]. The difference in the Met20 loop structures likely reflects the different binding states of the two enzyme preparations used; *Wb*DHFR is bound to folate and NADPH while the *Sm*DHFR structure is in the unbound apo state. The relatively high RMSD between the *Wb* and *Sm* DHFRs likely results from these differences. Other DHFRs from more evolutionarily distant organisms such as mouse DHFR and *Hs*DHFR share greater sequence identity (40–41%) with *Wb*DHFR but lower overall structural similarity (Table 2). In *Wb*DHFR, Arg-72 interacts with folate’s polyglutamic acid tail (Fig 4A and 4B). Similarly, *Sm*DHFR also has an arginine (Arg-67) in this position. In *Wb*DHFR, Glu-32 interacts with the pteridine ring of the substrate through hydrogen bonding; in *Sm*DHFR, this position is occupied by Asp-28, which likely forms similar interactions.

**Table 2:** The sequence identities and RMSD values for *Wb*DHFR bound to NADPH and folate compared with 13 other DHFR structures. Note: All structures are of the enzyme bound to cofactor NADPH and an antifolate except for *Sm*DHFR (PDB 3VCO).

Organism	PDB	Kingdom	%Sequence identity to *Wb*	RMSD to *Wb* (Å)	All Atom RMSD (Å)
*H*. *sapiens*	2W3M	Animalia	40	0.814	2.079 (1064 atoms)
*M*. *musculus*	3D80	Animalia	41	0.875	2.183 (1150 atoms)
*P*. *carinii*	1CD2	Fungi	33	0.893	4.458 (1130 atoms)
*A*. *flavus*	6DTC	Fungi	30	0.919	7.046 (905 atoms)
*M*. *tuberculosis*	1DF7	Bacteria	35	1.025	8.064 (947 atoms)
*E*. *coli*	4PDJ	Bacteria	32	1.043	3.463 (981 atoms)
*S*. *aureus*	3FRD	Bacteria	31	1.109	5.088 (903 atoms)
*M*. *avium*	2W3W	Bacteria	34	1.165	2.857 (964 atoms)
*M*. *profunda*	3IA4	Bacteria	35	1.202	3.773 (976 atoms)
*T*. *brucei*	3RG9	Excavata	38	1.212	7.307 (1074 atoms)
*C*. *albicans*	1AOE	Fungi	36	1.531	5.212 (1101 atoms)
*L*. *casei*	1LUD	Bacteria	24	1.560	2.930 (989 atoms)
*S*. *mansoni*	3VCO	Nematoda	32	1.324	3.331 (1126 atoms)

Given the similarities in folate binding motifs between the *Sm*DHFR and *Wb*DHFR, we hypothesize that novel antifolates with improved hydrophobic-binding capability could interact with both enzymes. As described in more detail below, there are differences in amino acid side chains between the *Hs*DHFR antifolate binding site and those of the two nematode DHFR structures described here, presenting an opportunity for the design of dual inhibitors for *Wb*DHFR and *Sm*DHFR that would be selective against *Hs*DHFR.

We further explored the amino acid sequence alignments, focusing specifically on the residues within the Met20 loop, a well-studied, catalytically significant structural element in the DHFR family of enzymes (Fig 2). The Met20 loop for *Wb*DHFR is most similar to the Met20 loop in *Ec*DHFR (residues 10–24) [51]. A seven amino acid long motif within the Met20 loop, containing two prolines that are spaced three residues apart (PWXLPAX) is clearly present in *Wb*DHFR (Fig 2 and Table A in S1 Text). The same motif in *Sm*DHFR is also seven amino acids long but contains only one proline (**P**WKIKKD). Previous literature has suggested that longer motif length is associated with lower conformational flexibility of the Met20 loop, preventing large-scale opening and closing loop motions [23] and thus influencing the catalytic cycle [52]. Based on these observations, we predict that the Met20 loops of both *Sm*DHFR and *Wb*DHFR undergo conformational motions during the catalytic cycle. In contrast, *Hs*DHFR has a longer Met20 loop with 8 residues in the motif (**P**W**PP**LRNE) and does not undergo closed to occluded transitions [23, 51].

The sequence of the Met20 loop of *Wb*DHFR compared to other DHFR orthologs (Fig 2) is largely similar with the two exceptions being Met-25 and Phe-28. Met-25, which is replaced by either leucine or isoleucine in other DHFR orthologs, makes nominal contacts with folate in both DHFR models from *E*. *coli* and *W*. *bancrofti*. Phe-28, a residue that is not conserved in other orthologs, does not make any contacts with folate but points toward the active site and could conceivably make hydrophobic contacts with a longer antifolate. Also, the Met20 loop is less flexible based on root mean square fluctuations (RMSFs) from the MD simulations for *Hs*DHFR when compared to *Wb*DHFR (Fig G in S1 Text). A more flexible loop, as observed for *Wb*DHFR, may be able to sample more conformations and increase overall interactions with a longer antifolate that expands into this region.

### Molecular docking of antifolate compounds

In previous studies, we tested antifolates as potential inhibitors of *Wb*DHFR; methotrexate (K_I_ = 0.7 ± 0.1 nM) and structurally similar aminopterin (K_I_ = 2.1 ± 0.5 nM) were found to be the most potent inhibitors of those that were tested [22]. Other antifolates, including trimethoprim and pyrimethamine were low micromolar inhibitors. The crystal structure published here allowed us to extend these studies through molecular docking.

**Fig 5:**
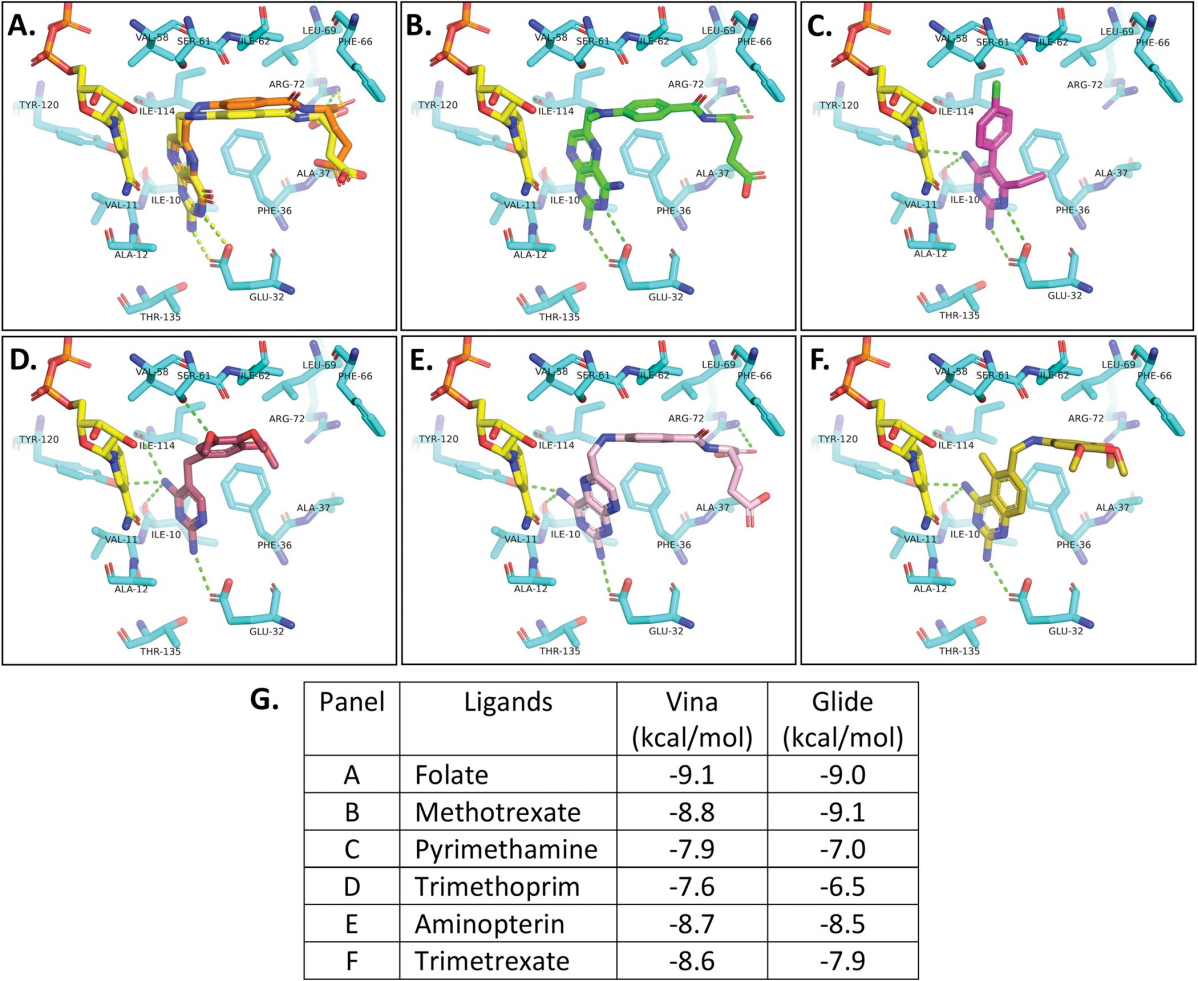
Results of molecular docking of folate and antifolates into the *Wb*DHFR active site using AutoDock Vina. Hydrogen bonds between the co-crystallized folate (A) and *Wb*DHFR (PDB: 8E4F) are denoted as yellow dashes. Green dashes indicate potential hydrogen bonds between the docked antifolates and *Wb*DHFR. In panel A, co-crystallized folate (yellow) and docked folate (orange) exhibit comparable conformations (See Fig A in S1 Text for structures). Larger inhibitors that resemble folate, i.e. methotrexate (green; B) and aminopterin (pink; E) and smaller antifolates pyrimethamine (magenta; C), trimethoprim (dark red, D), and trimetrexate (yellow, F), are shown. The energy scores from AutoDock Vina and Schrödinger Glide for docked ligands are shown in G.

The docking poses of antifolates obtained from Vina and Glide were largely similar for a given antifolate (Fig H in S1 Text). The 2D ligand interaction diagrams for Glide and Vina also showed similar interactions (Figs I and J in S1 Text). VINA docking results predicted that folate analogs methotrexate and aminopterin have similar conformations and docking scores, -8.8 and -8.7 kcal/mol, respectively (Fig 5). These scores are also similar to the value predicted for folate (-9.1 kcal/mol). The Glide output energies for methotrexate (-9.1 kcal/mol) and aminopterin (-8.5 kcal/mol) also supported the VINA docking predictions (Table B in S1 Text). Methotrexate and aminopterin contain a modified pteridine ring, central hydrophobic linker, and polyglutamic acid tail, similar to the substrate DHF (Fig A in S1 Text). Here, we classified the traditional antifolates as having either a “longer” or “shorter” linker based on the number of “linker” atoms between pyrimidine/pteridine and polyglutamic acid pharmacophores. This classification includes atoms in the para-benzoic motif (8 atoms) but does not count the atoms in the pteridine ring or polyglutamic acid tail. Overall, the “shorter” linkers consist of 0–1 atoms while “longer” linkers are composed of >7 atoms. The longer linker in methotrexate and aminopterin is positioned to interact with nonpolar amino acids Phe-36, Leu-69, and Met-33 in the hydrophobic core of the active site of *Wb*DHFR. Pyrimethamine has no such linker and trimethoprim has only a short linker consisting of one CH_2_ group (Fig A in S1 Text). The docking predictions shown in Fig 5 suggested that these smaller antifolates made fewer interactions with the hydrophobic core of the enzyme active site.

The docking results provide insight for *Wb*DHFR-antifolate interactions but there are several limitations to these models. The docking scores only provide relative estimates of binding affinities and must be validated through binding assays. We observed some correlations between the docking scores and affinity measurements. For example, the experimentally determined K_I_ values for methotrexate and pyrimethamine (0.7± 0.1 nM and 15 ± 6 μM) qualitatively correlated with the Vina (-8.8 kcal/mol and -7.9 kcal/mol) and Glide (-9.1 kcal/mol and -7.0 kcal/mol) estimated binding energies. The scoring functions for Vina and Glide are similar and rely on summing contributions of hydrophobic and hydrophilic interactions. The score differences may be due to the different force fields, OPLS and AMBER, which Glide and Vina use, respectively. While our docking comparison suggests that the OPLS force field works better for the *Wb*DHFR structure, the docking scores only provide relative estimates of binding. The predicted absolute energies from molecular docking may not reflect experimentally determined K_I_ values due to assumptions such as rigid-body protein receptor, no solvation, and simplified general scoring [53, 54]. However, the trend in the predicted binding energies from Vina and Glide docking programs agree that antifolates with longer linkers can make more contacts with *Wb*DHFR compared to shorter linker antifolates.

### MD simulations

The MD simulations provided additional insights into the binding interactions between *Wb*DHFR and the antifolates. System equilibration of the *Wb*DHFR and *Hs*DHFR simulations was confirmed through plotting RMSD over time (Figs K and L in S1 Text). Similar to the crystal structure, the MD simulations showed strong interactions between folate and Glu-32, Ile-10, and Arg-72. Additional interactions included the stacking interaction with Phe-36 and several van der Waals contacts with hydrophobic residues. The simulations provided the relative fraction or percentage of time during which the interaction was observed over 10 nanoseconds; strong interactions are those that were observed >50% of the time in the simulation.

**Fig 6:**
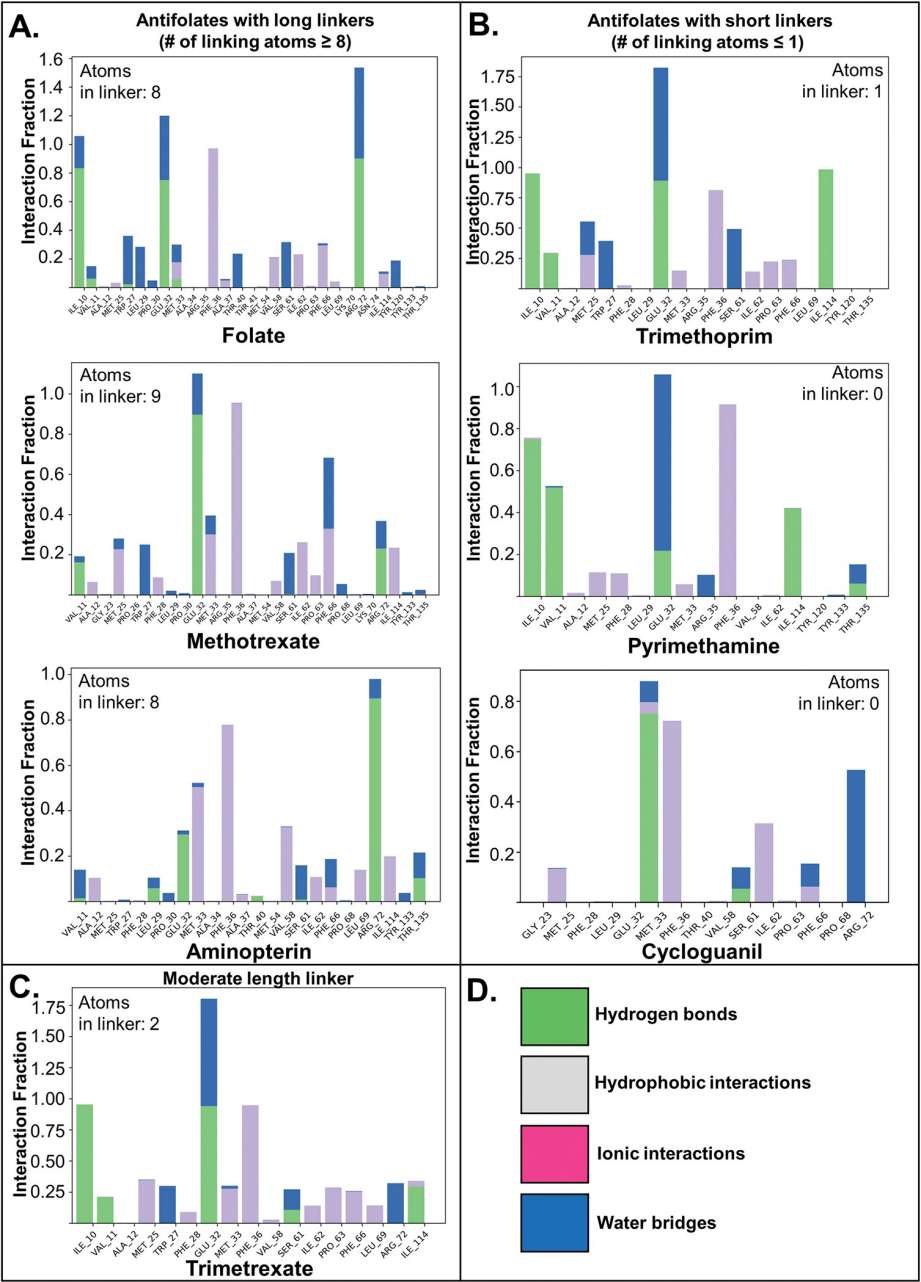
Interaction fractions depicted for *Wb*DHFR and various antifolate types. Antifolates with longer (A), shorter (B), or moderate length linkers (C) were grouped to show hydrophobic binding patterns. Residues interacting with the antifolate are listed on the x-axis; interaction fraction on the y-axis describes the fraction of time each interaction is observed in the simulation (fraction of 1.0 = interaction is observed in 100% of snapshots). In cases where the interaction fraction is >1.0, the residues (or atoms) may be involved in more than one interaction. Common interactions are color-coded including hydrogen bonds (green), hydrophobic contacts (purple), ionic bonds (pink), and water bridges (blue) (D). Comparison of interaction fractions shows that the long linker antifolates make more hydrophobic contacts.

The crystal structure, K_I_ data, and molecular docking scores suggest that antifolates with longer linkers have better affinity for *Wb*DHFR. When comparing total protein-ligand contacts for all MD simulations, we found that antifolates with longer linkers make more overall hydrophobic contacts in the simulation compared to antifolates with shorter linkers. Fig 6 shows a comparison of the interaction fractions for key residues for simulations conducted for *Wb*DHFR and all antifolates. When comparing the interaction fractions, antifolates with longer linkers made more hydrophobic contacts for longer fractions or periods of time in the simulation.

MD simulations were also used to compare the *Wb*DHFR and *Hs*DHFR Met20 loop regions. Using average root mean square fluctuation (RMSF) values for residues within the Met20 loop, we found that *Wb*DHFR undergoes more fluctuations compared to *Hs*DHFR (Fig G in S1 Text). This finding supports the hypothesis that *Hs*DHFR adopts a closed conformation, while *Wb*DHFR undergoes conformational motions during the catalytic cycle.

### Insights for rational design of *Wb*DHFR inhibitors

**Fig 7:**
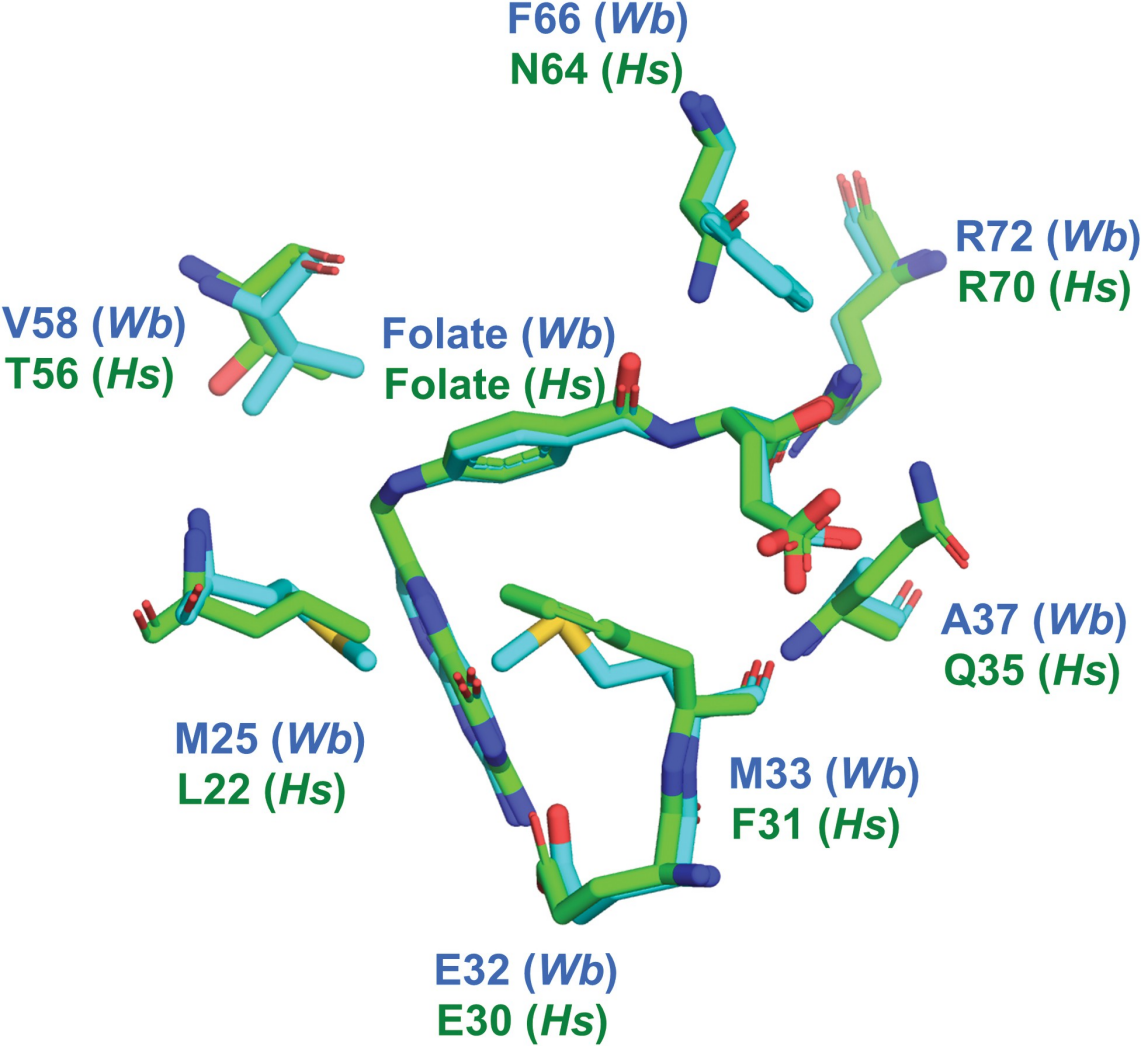
The folate binding sites of the NADPH and folate bound DHFR structures from *Wb* (cyan; PDB 8E4F) and human (green; PDB 2W3M) were aligned using PyMOL. Key differences in the binding pocket are shown.

Experimental data have demonstrated that traditional antifolate substrate analogs can inhibit *Wb*DHFR. Subsequent molecular docking predictions using the *Wb*DHFR X-ray structure corroborated this finding and revealed potential strategies for drug design. As described below, there are amino acid side chain differences between the folate binding sites of *Hs*DHFR and *Wb*DHFR (Fig 7), suggesting opportunities for developing *Wb*DHFR selective inhibitors. There are several differences between the folate binding sites of *Wb*DHFR and *Hs*DHFR (PDB 2W3M). Docking the antifolates to *Hs*DHFR and *Wb*DHFR with Glide and Vina shows that methotrexate, a *Hs*DHFR-tailored inhibitor, is predicted to bind with high affinity to both enzymes (Table C in S1 Text). Glide predicts that trimetrexate has a higher affinity for *Wb*DHFR than *Hs*DHFR. Two hydrophilic residues that form side chain hydrogen bonds to folate in the human structure (Gln-35 and Asn-64) have hydrophobic counterparts in the *Wb*DHFR structure (Ala-37 and Phe-66) (Fig 7). One similarity between the *Wb*DHFR and *Sm*DHFR is that they share active site hydrophobic residues unique to these nematode orthologs such as Met-33 and Phe-66 in *Wb*DHFR and the corresponding Met-29 and Phe-61 in *Sm*DHFR. Therefore, small molecules with hydrophobic linker motifs may serve as dual inhibitors for both *Sm*DHFR and *Wb*DHFR enzymes while lacking affinity for the human enzyme.

An antifolate where the carbonyl O atom (Fig A in S1 Text) has been replaced with a nonpolar functional group or compounds with longer and more hydrophobic polyglutamic acid tails could be more selective for *Wb*DHFR over *Hs*DHFR. Based on the docking predictions shown in Fig 5 an effective strategy to develop antifolates targeting *Wb*DHFR could exploit the side chains of residues Glu-32, Arg-72, Tyr-120, and Ser-61 and the backbone carbonyls of Ile-10 and Ile-114.

The active site of *Wb*DHFR contains hydrophobic regions consisting of residues Phe-66, Ala-37, and Val-58 (Fig 7). These residues may also be used to anchor aliphatic substitutions in novel antifolates, since they are not present in the human ortholog, in which hydrophilic side chains occupy these positions. The predictions shown here and in previous studies have noted the importance of linker length and hydrophobic moieties connecting the diaminopyrimidine pharmacophore in the design of antifolates [44, 55, 56]. In further support of this importance, our docking models for both trimetrexate and trimethoprim (both containing a trimethoxyphenyl functional group) predict a more negative docking score for trimetrexate (-8.6 kcal/mol) than for the shorter trimethoprim (-7.6 kcal/mol). This may again be due to the linker length of trimetrexate and its subsequent ability to extend further, thus taking advantage of additional hydrophobic residues such as Leu-69 and Phe-66 (Fig 5). We determined the IC_50_ value of trimetrexate experimentally to be 0.49 ± 0.16 μM (Fig M in S1 Text), which lies between the previously determined IC_50_ values for trimethoprim and methotrexate (83 ± 25 μM and 0.018 ± 0.003 μM, respectively). The docking results and the biochemical data suggest that interactions with the more hydrophobic residues in the active site of *Wb*DHFR are important for efficient inhibition.

We now have the crystal structure of *Wb*DHFR with folate and NADPH bound in the active site. Our studies were limited to mostly computational methods when exploring the linker length of effective antifolate inhibitors for *Wb*DHFR. Therefore, future experiments may involve solving the crystal structure of *Wb*DHFR bound to antifolates with different linker lengths. In summary, inspection of the *Wb*DHFR crystal structure, docking predictions, and results from MD simulations suggest that novel antifolates designed to contain a central hydrophobic linker directed to interact with the now well-established *Wb*DHFR target regions could help make antifolate therapy a reality in the treatment of filarial nematode infections.

## Supporting information

S1 Text **Fig A.** Structures and atom labelling of *Wb*DHFR ligands folate, NADPH and antifolates methotrexate (MTX), pyrimethamine (PYR), trimethoprim (TMP), aminopterin (AMP), trimetrexate (TMX), and dihydrofolate. Structures were obtained from the ligand summary pages in the Protein Data Bank. **Fig B.** (A) SDS-PAGE (4–20% polyacrylamide gel) of purification fractions for *Wb*DHFR. MWM–molecular weight markers; MTA–methotrexate agarose; FT–flow through. (B) Microscopic image of *Wb*DHFR crystals grown by sitting-drop vapor diffusion. **Fig C.** Omit electron density for active site residues of *Wb*DHFR, folate, and NADPH. Electron density is contoured to 1.0 sigma. **Fig D.** Distances in Å between atoms of folate (yellow) and residues of *Wb*DHFR (cyan) that form hydrogen bonds are shown. The two amino acids responsible for forming hydrogen bonds with folate, Glu-32 and Arg-72, are shown as sticks. The predicted distance of the hydride transfer from NADPH to folate is also shown. The distances were measured in PyMOL. **Fig E.** Alignment of ternary structures of *Hs*DHFR and *Wb*DHFR demonstrates the similarity of ligand conformations for both folate and NADPH. The ternary structure for *Hs*DHFR (PDB: 2W3M, green) was obtained from the PDB and was aligned with *Wb*DHFR in PyMOL. The conformation of folate for both *Wb*DHFR and *Hs*DHFR is very similar; however, the adenine moiety in NADPH is differently positioned in the two structures. **Fig F.** Alignment of *Sm*DHFR apoenzyme (PDB: 3VCO, magenta) and the *Wb*DHFR ternary structure (PDB: 8E4F, cyan) done in PyMOL. Folate and NADPH from *Wb*DHFR are shown in yellow. The Met20 of the ternary *Wb*DHFR structure is in the closed conformation while the Met20 loop of *Sm*DHFR is in the disordered conformation. **Table A:** Met20 loop, B-factor, and steady state parameter data for four DHFR homologs. **Fig G.** Comparison of root mean square fluctuation (RMSF) values for residues within the Met20 loop region (5–35) in Angstroms: *Wb*DHFR (blue) and *Hs*DHFR (red). *Wb*DHFR shows more fluctuation in the Met20 loop region whereas *Hs*DHFR shows less fluctuation. **Fig H.** Alignment of docking poses of antifolates obtained from Glide and Vina. The docking poses of the antifolates from Glide (green) and Vina (violet) with the receptor *Wb*DHFR (cyan). The NADPH cofactor is seen as yellow. **Table B:** Summary of interactions observed in molecular docking models obtained from both Autodock and Glide. **Fig I.** Ligplot images generated for docking models obtained from Autodock Vina. **Fig J.** Ligand interaction diagrams generated for docking models obtained from Glide. A) *Wb*DHFR-Folic Acid; B) *Wb*DHFR-Methotrexate; C) *Wb*DHFR-Aminopterin; D) *Wb*DHFR-Pyrimethamine; E) *Wb*DHFR-Trimethoprim; F) *Wb*DHFR-Trimetrexate. See Fig 4 for legend. **Fig K**: Root mean square deviation (RMSD) values per time in nanoseconds (ns) for all *Wb*DHFR molecular dynamics (MD) simulations conducted at 10 ns. RMSD values for the Cα backbone of *Wb*DHFR (blue) and ligand (red) show equilibration by the end of 10 ns. According to Desmond Simulation Interaction Reports, changes of the order of 1–3 Å for the Cα backbone are acceptable for small, globular proteins such as *Wb*DHFR. **Fig L.** Root mean square deviation (RMSD) values per time in nanoseconds (ns) for the *Hs*DHFR molecular dynamics (MD) simulations conducted at 10 ns. RMSD values for the Cα backbone of *Wb*DHFR (blue) and ligand (red) show equilibration by the end of 10 ns. According to Desmond Simulation Interaction Reports, changes of the order of 1–3 Å for the Cα backbone are acceptable for small, globular proteins such as *Wb*DHFR. **Table C.** Comparison of docking scores of antifolates to *Hs*DHFR and *Wb*DHFR using both Glide and Vina. **Fig M.** Evaluation of trimetrexate as an inhibitor of *Wb*DHFR. Enzyme activity plot of *Wb*DHFR against a titration of trimetrexate from 100 μM down to 1.6 nM (see method above). The resulting plot is the average of four independent measurements with error bars representing standard error. The data was fitted to a sigmoidal curve in KaleidaGraph software V 4.5.4 where an IC_50_ of 491 ± 161 nM was observed. Using the Cheng-Prusoff equation and a Michaelis-Menten constant of 3.7 μM, the K_I_ was calculated to be 33 ± 11 nM.
(DOCX)
