# Supplementary material for: Crystal structure of dihydrofolate reductase from the filarial nematode W. bancrofti in complex with NADPH and folate
Source: PLoS Negl Trop Dis. 2023 Apr 27;17(4):e0011303. doi: 10.1371/journal.pntd.0011303 (PMC10191325; doi:10.1371/journal.pntd.0011303)
Supplement: S1 Text — Fig A. Structures and atom labelling of WbDHFR ligands folate, NADPH and antifolates methotrexate (MTX), pyrimethamine (PYR), trimethoprim (TMP), aminopterin (AMP), trimetrexate (TMX), and dihydrofolate. Structures were obtained from the ligand summary pages in the Protein Data Bank. Fig B. (A) SDS-PAGE (4–20% polyacrylamide gel) of purification fractions for WbDHFR. MWM–molecular weight markers; MTA–methotrexate agarose; FT–flow through. (B) Microscopic image of WbDHFR crystals grown by sitting-drop vapor diffusion. Fig C. Omit electron density for active site residues of WbDHFR, folate, and NADPH. Electron density is contoured to 1.0 sigma. Fig D. Distances in Å between atoms of folate (yellow) and residues of WbDHFR (cyan) that form hydrogen bonds are shown. The two amino acids responsible for forming hydrogen bonds with folate, Glu-32 and Arg-72, are shown as sticks. The predicted distance of the hydride transfer from NADPH to folate is also shown. The distances were measured in PyMOL. Fig E. Alignment of ternary structures of HsDHFR and WbDHFR demonstrates the similarity of ligand conformations for both folate and NADPH. The ternary structure for HsDHFR (PDB: 2W3M, green) was obtained from the PDB and was aligned with WbDHFR in PyMOL. The conformation of folate for both WbDHFR and HsDHFR is very similar; however, the adenine moiety in NADPH is differently positioned in the two structures. Fig F. Alignment of SmDHFR apoenzyme (PDB: 3VCO, magenta) and the WbDHFR ternary structure (PDB: 8E4F, cyan) done in PyMOL. Folate and NADPH from WbDHFR are shown in yellow. The Met20 of the ternary WbDHFR structure is in the closed conformation while the Met20 loop of SmDHFR is in the disordered conformation. Table A: Met20 loop, B-factor, and steady state parameter data for four DHFR homologs. Fig G. Comparison of root mean square fluctuation (RMSF) values for residues within the Met20 loop region (5–35) in Angstroms: WbDHFR (blue) and HsDHFR (red). WbDHFR shows more fluctua [file pntd.0011303.s001.docx]

**Supporting information**

**
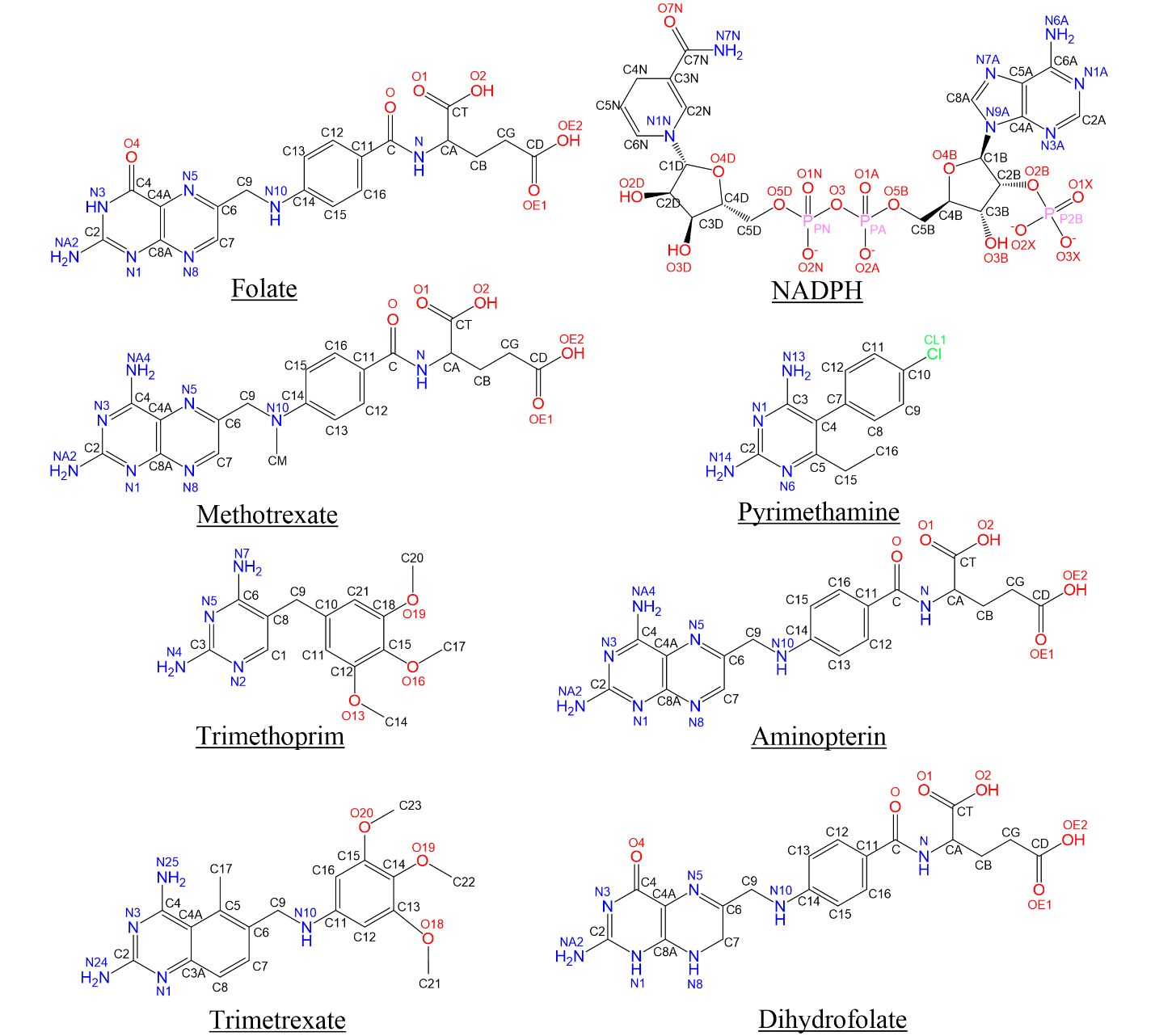
**

**Fig A.** Structures and atom labelling of *Wb*DHFR ligands folate, NADPH and antifolates methotrexate (MTX), pyrimethamine (PYR), trimethoprim (TMP), aminopterin (AMP), trimetrexate (TMX), and dihydrofolate. Structures were obtained from the ligand summary pages in the Protein Data Bank.


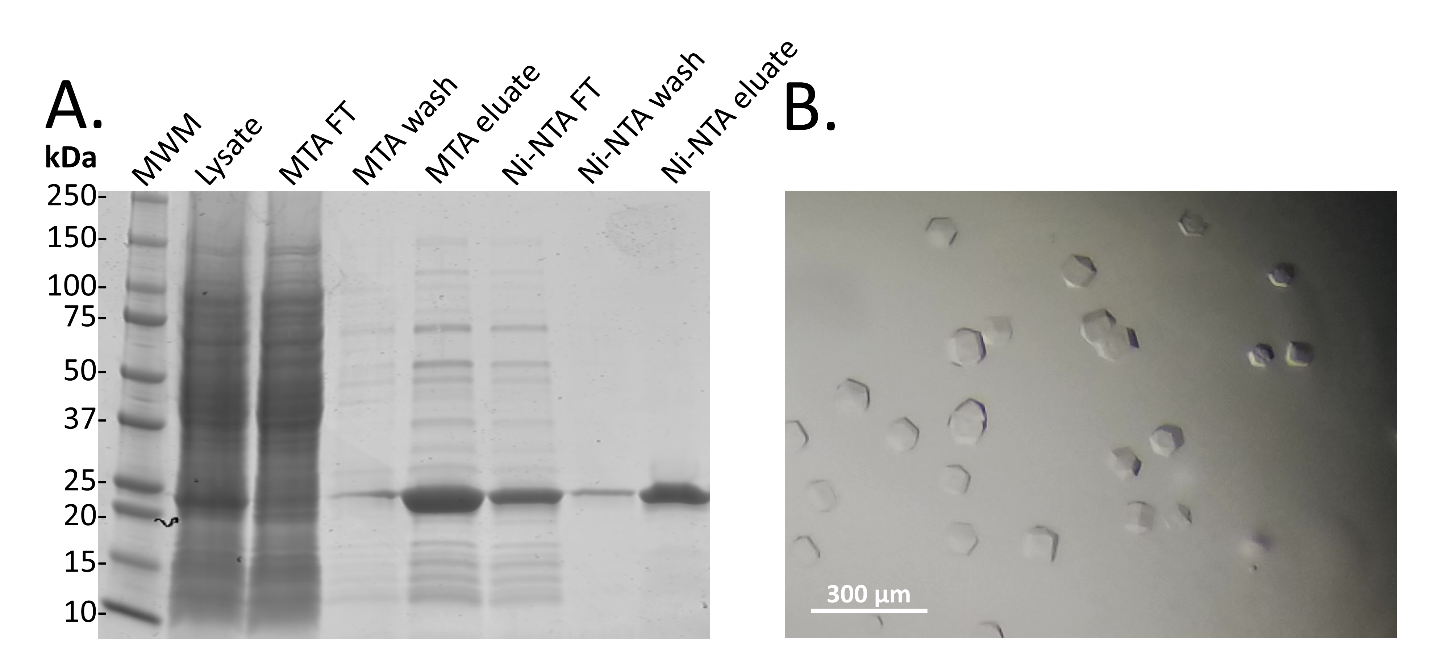


**Fig B.** (A) SDS-PAGE (4-20% polyacrylamide gel) of purification fractions for *Wb*DHFR. MWM – molecular weight markers; MTA – methotrexate agarose; FT – flow through. (B) Microscopic image of *Wb*DHFR crystals grown by sitting-drop vapor diffusion.


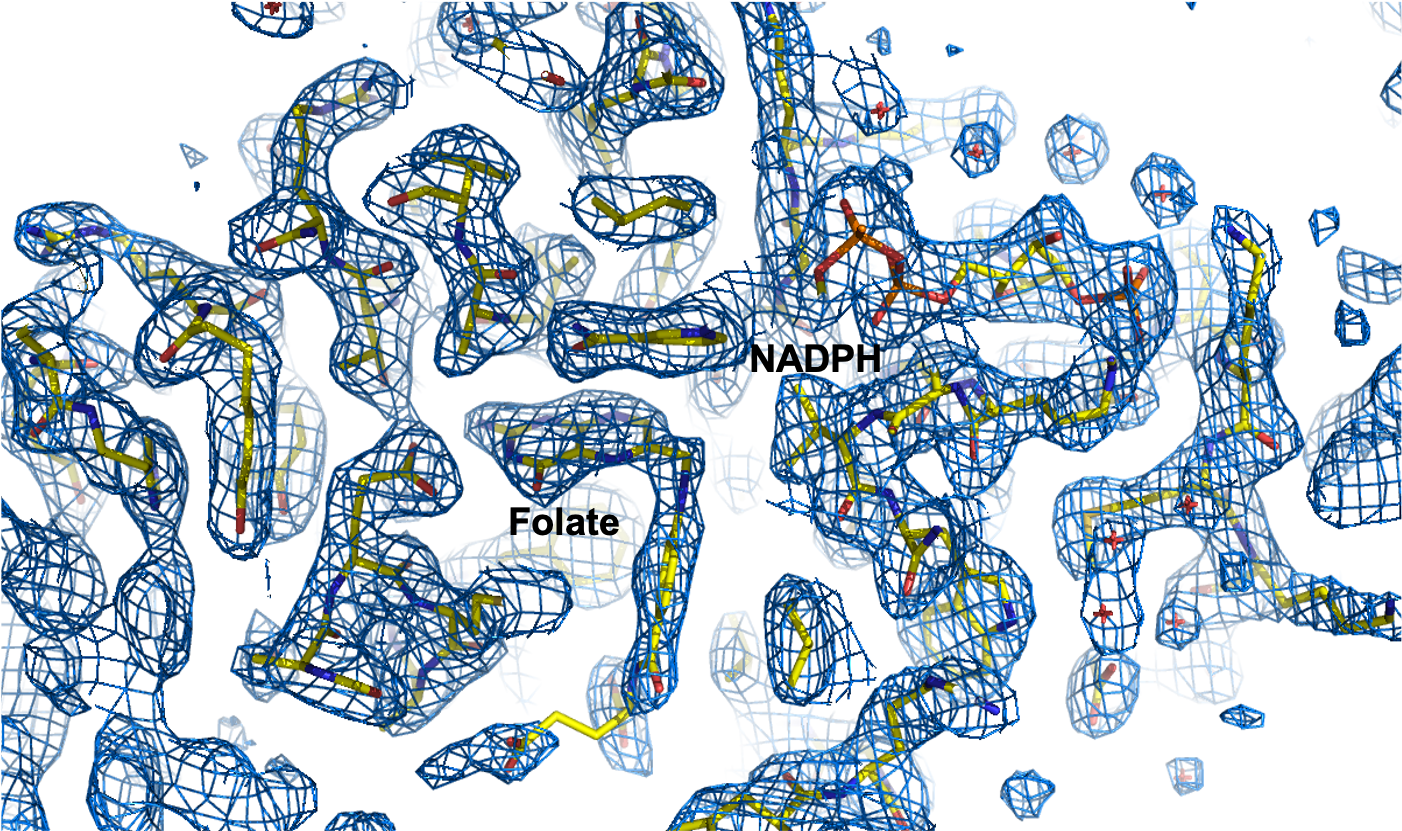


**Fig C.** Omit electron density for active site residues of *Wb*DHFR, folate, and NADPH. Electron density is contoured to 1.0 sigma.

**
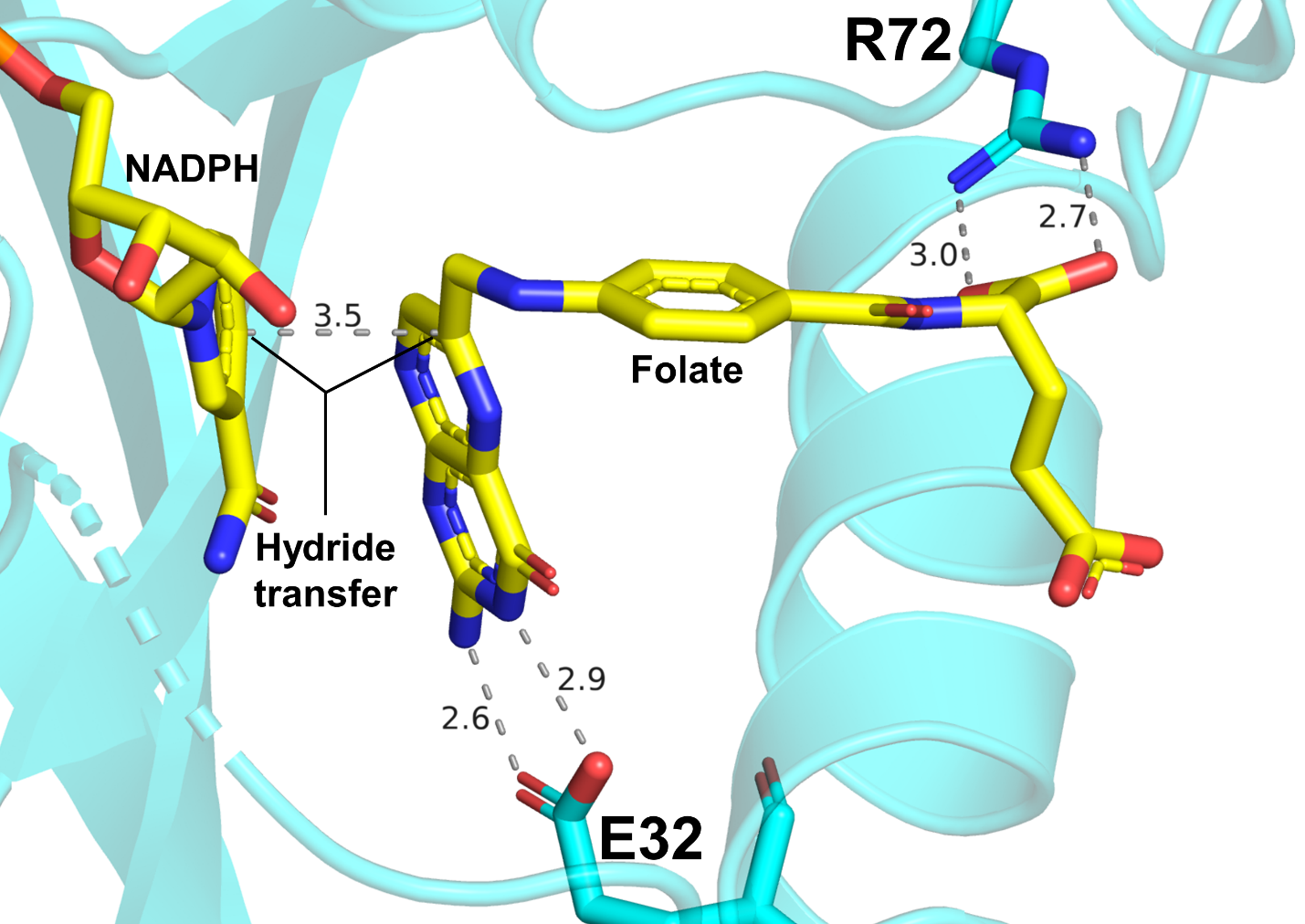
**

**Fig D.** Distances in Å between atoms of folate (yellow) and residues of *Wb*DHFR (cyan) that form hydrogen bonds are shown. The two amino acids responsible for forming hydrogen bonds with folate, Glu-32 and Arg-72, are shown as sticks. The predicted distance of the hydride transfer from NADPH to folate is also shown. The distances were measured in PyMOL.

**
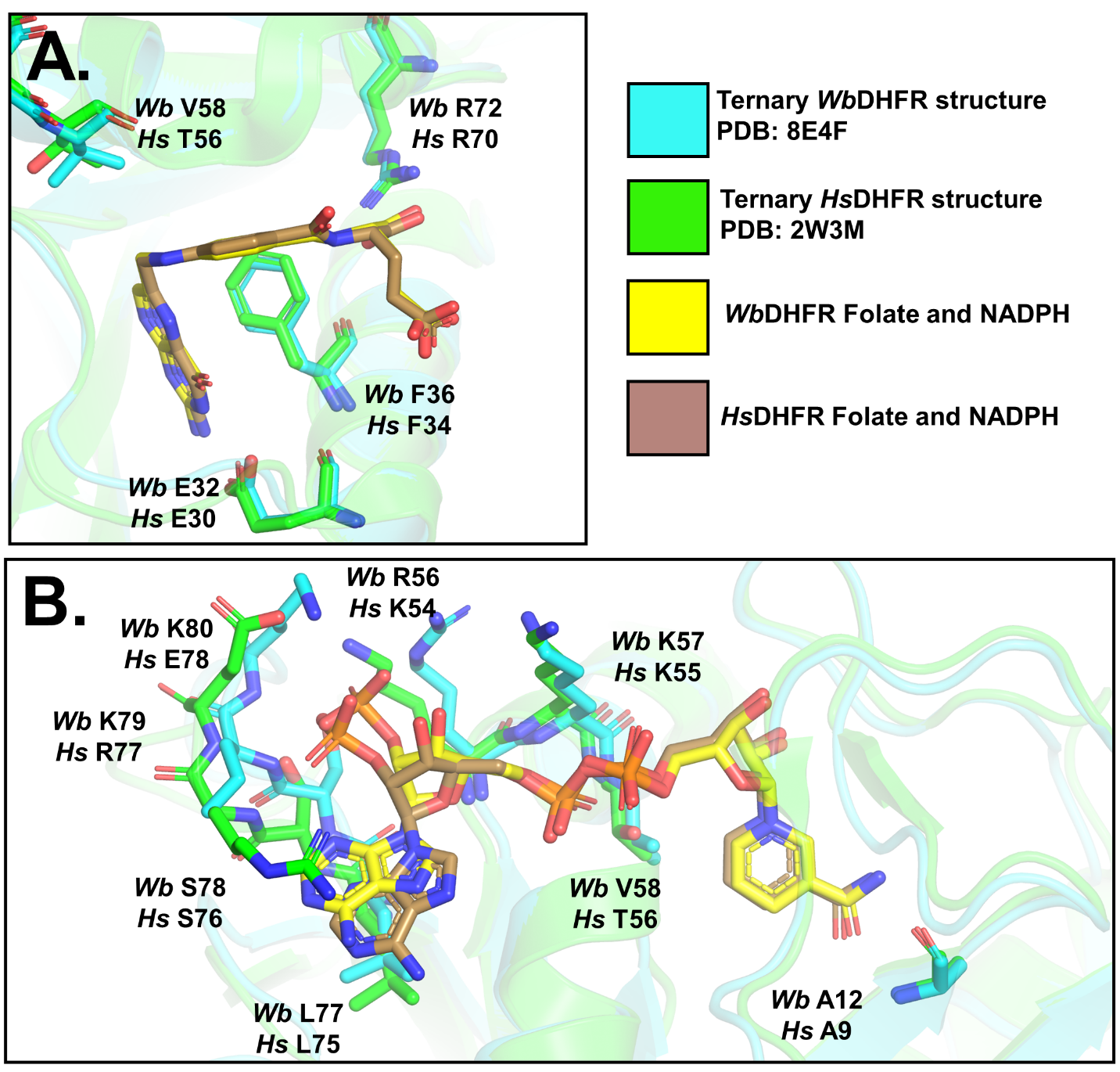
**

**Fig E.** Alignment of ternary structures of *Hs*DHFR and *Wb*DHFR demonstrates the similarity of ligand conformations for both folate and NADPH. The ternary structure for *Hs*DHFR (PDB: 2W3M, green) was obtained from the PDB and was aligned with *Wb*DHFR in PyMOL. The conformation of folate for both *Wb*DHFR and *Hs*DHFR is very similar; however, the adenine moiety in NADPH is differently positioned in the two structures.


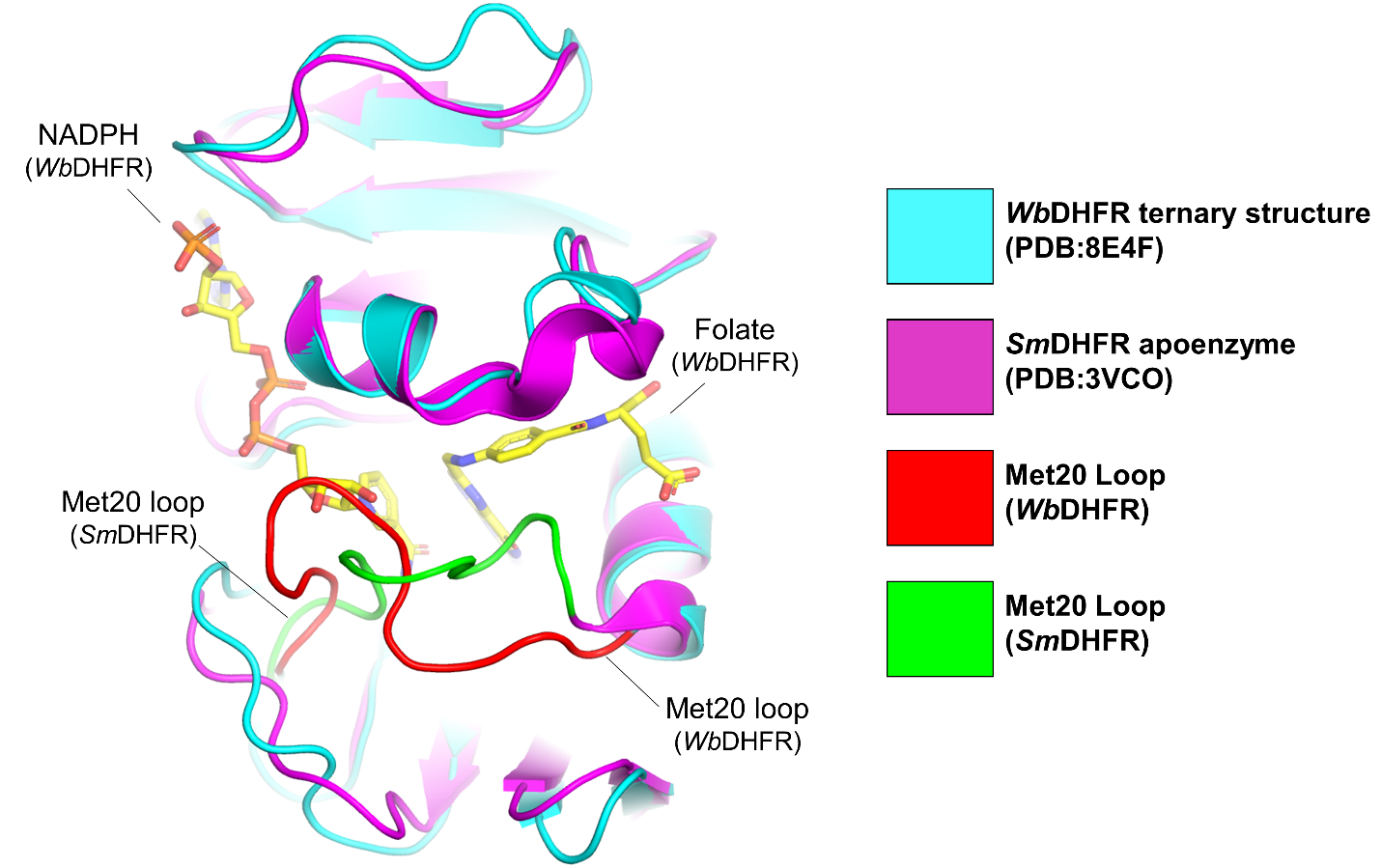


**Fig F.** Alignment of *Sm*DHFR apoenzyme (PDB: 3VCO, magenta) and the *Wb*DHFR ternary structure (PDB: 8E4F, cyan) done in PyMOL. Folate and NADPH from *Wb*DHFR are shown in yellow. The Met20 of the ternary *Wb*DHFR structure is in the closed conformation while the Met20 loop of *Sm*DHFR is in the disordered conformation.

**Table A:** Met20 loop, B-factor, and steady state parameter data for four DHFR homologs.

| Species/  DHFR | Met20 Loop Motif Sequence (# residues) | Average B-factors for Met20 loop residues (Å^2^) | K_M_ DHF  (μM) | *k_cat_* DHF  (s^-1^) | Kcat/KM (μM s^-1^) | Reference |
| --- | --- | --- | --- | --- | --- | --- |
| *Wb*DHFR | **P**WFL**P**AE (7) | 25.97 | 3.7 | 7.4 | 2 | *PloS one* 2018, 13 (5), e0197173. |
| *Ec*DHFR | **P**WNL**P**AD (7) | 35.82 | 0.7 | 12 | 17.1 | *Biochemistry* (2010) 49: 195-206. |
| *Sm*DHFR | **P**WKIKKD (7) | 49.88 | 26.4 | 35.9 | 1.36 | *Acta Tropica* (2017) 170: 190-196. |
| *Hs*DHFR | **P**W**PP**LRNE (8) | 20.18 | 36 | 40 | 1.1 | *Protein Expr. Purif.*(1993) 4, 16–23. *Mol. Biochem. Parasitol.* (2001) 113, 241–249. |

**
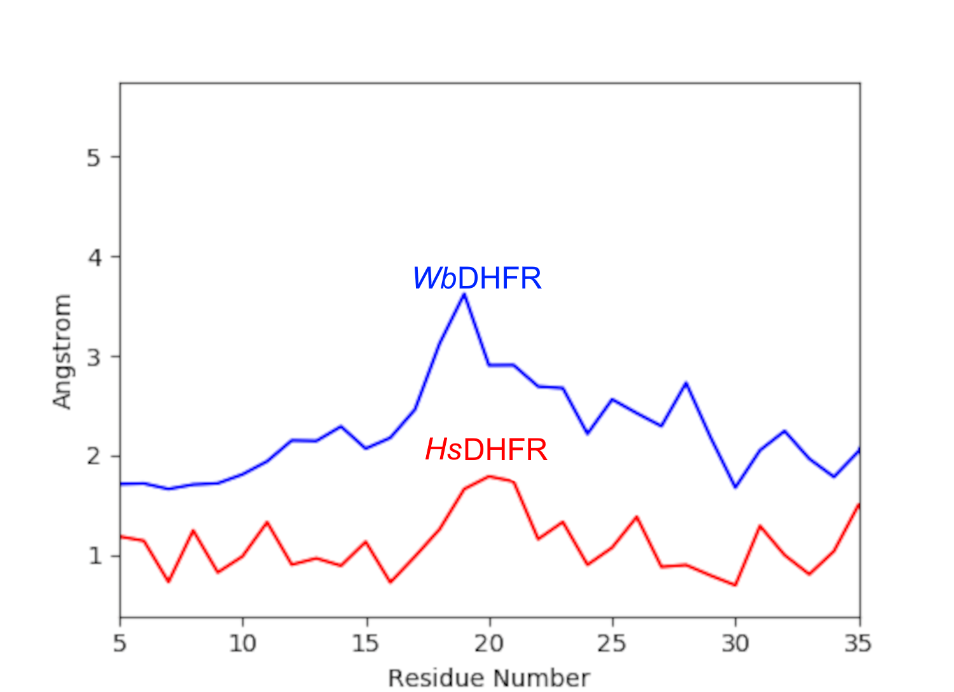
**

**Fig G.** Comparison of root mean square fluctuation (RMSF) values for residues within the Met20 loop region (5-35) in Angstroms: *Wb*DHFR (blue) and *Hs*DHFR (red). *Wb*DHFR shows more fluctuation in the Met20 loop region whereas *Hs*DHFR shows less fluctuation.

**
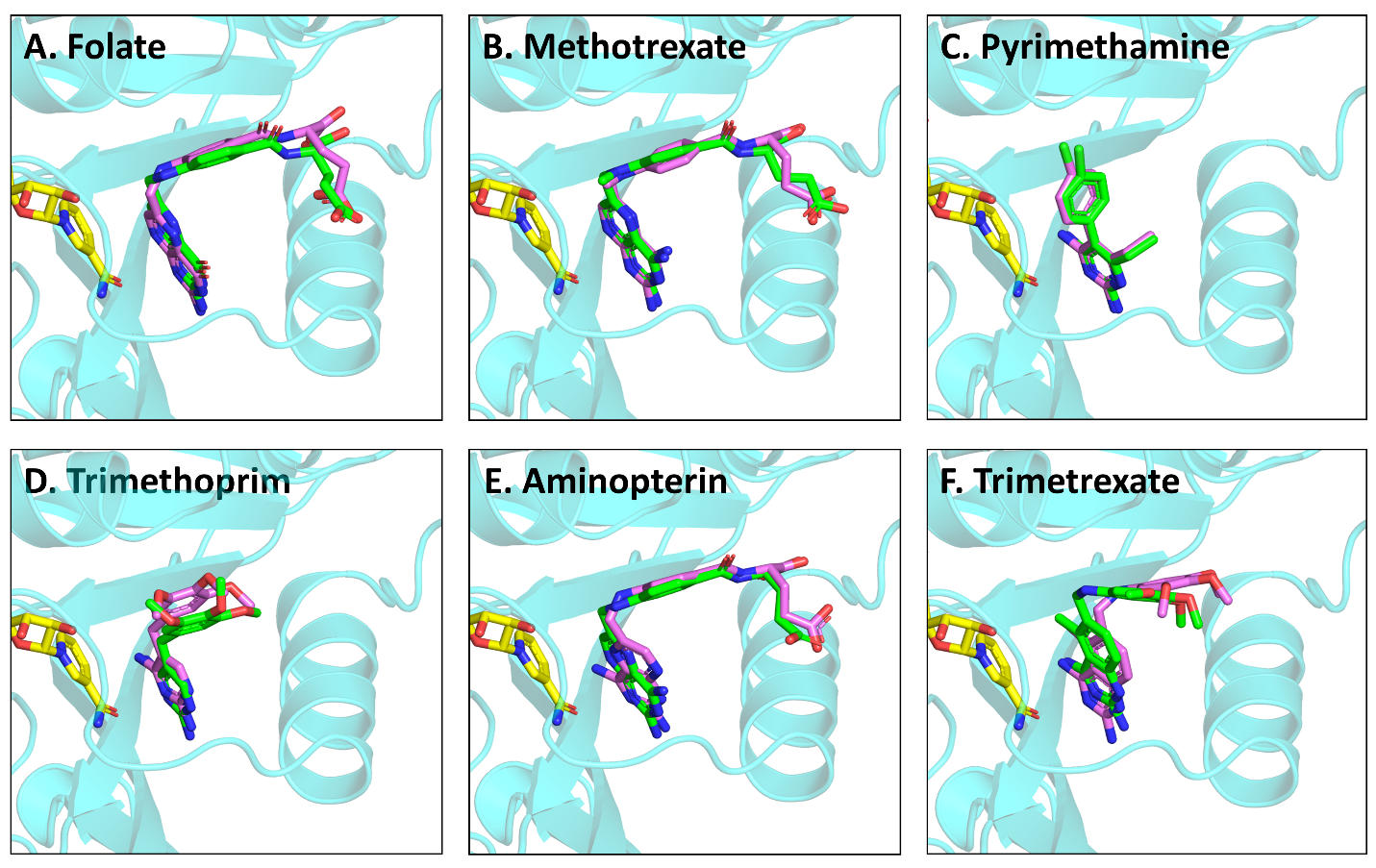
**

**Fig H.** Alignment of docking poses of antifolates obtained from Glide and Vina.  The docking poses of the antifolates from Glide (green) and Vina (violet) with the receptor *Wb*DHFR (cyan). The NADPH cofactor is seen as yellow.

**Table B:** Summary of interactions observed in molecular docking models obtained from both Autodock and Glide.

| Docking Model WbDHFR:Ligand  (Docking Score) | Docking Scores in Autodock and Glide (kcal/mol) | Hydrogen Bonds with WbDHFR (Antifolate group-Residue) | Stacking Interactions with *Wb*DHFR | Van der Waals contacts with *Wb*DHFR |
| --- | --- | --- | --- | --- |
| Folate | Autodock =-9.1  Glide = -9.0 | NH (pteridine N3)-Glu32  NH_2_ (pteridine NA2)-Glu32  O1 (glutamate)-Arg72  O2 (glutamate OH)-Arg72 | Pteridine-Phe36 | Ile10, Val11, Ala12, Met25, Phe28, Met33, Ala34, Phe36, Ala37, Val58, Ser61, Ile62, Pro63, Phe66, Leu69, Ile114, Tyr120, Thr135, NADPH |
| Methotrexate | Autodock = -8.8  Glide = -9.1 | NH_2_ (pteridine NA2)-Glu32  O1 (glutamate)-Arg72  N (pteridine N3)-Glu32  N (pteridine N8)-Ile10 | Pteridine-Phe36 | Ile10, Val11, Ala12, Met25, Met33, Ala34, Phe36, Ala37, Val58,  Ser61, Ile62, Pro63, Phe66, Leu69, Ile114, Tyr120, Thr135, NADPH |
| Aminopterin | Autodock = -8.7  Glide = -8.5 | NH_2_ (pteridine NA2)-Glu32  O1 (glutamate)-Arg72  NH_2_ (pteridine NA4)-Ile10  NH_2_ (pteridine NA4)-Tyr120 |  | Ile10, Val11, Ala12, Met25, Phe28, Met33, Ala34, Phe36, Ala37, Val58, Ser61, Ile62, Pro63, Phe66, Leu69, Ile114, Tyr120, Thr135, NADPH |
| Pyrimethamine | Autodock = -7.9  Glide = -7.0 | NH_2_ (pyrimidine N14)-Glu32  NH_2_ (pyrimidine N13)-Ile10  N (pyrimidine N6)-Glu32  NH_2_ (pyrimidine N13)-Tyr120 | Cl-Phenyl-Phe36 | Ile10, Val11, Ala12, Met25, Met33, Phe36, Val58, Ser61, Ile62, Ile114, Ty212, Thr135, NADPH |
| Trimethoprim | Autodock = -7.6  Glide = -6.2 | NH_2_ (pyrimidine N4)-Glu32  NH_2_ (pyrimidine N7)-Ile10  NH_2_ (pyrimidine N7)-Ile114  NH_2_ (pyrimidine N7)-Tyr120 |  | Ile10, Val11, Ala12, Met25, Met33, Phe36, Ser61, Ile62, Pro63, Ile114, Ty2120, Thr135  NADPH |
| Trimetrexate | Autodock = -8.6  Glide = -7.9 | NH_2_ (pyrimidine N24)-Glu32  NH_2_ (pyrimidine N25)-Ile10  NH_2_ (pyrimidine N25)-Ile114  NH_2_ (pyrimidine N25)-Tyr120 |  | Ile10, Val11, Ala12, Met25, Phe28, Met33, Phe36, Val58, Ser61, Ile62, Pro63, Ile114, Tyr212, Thr135, NADPH |

**
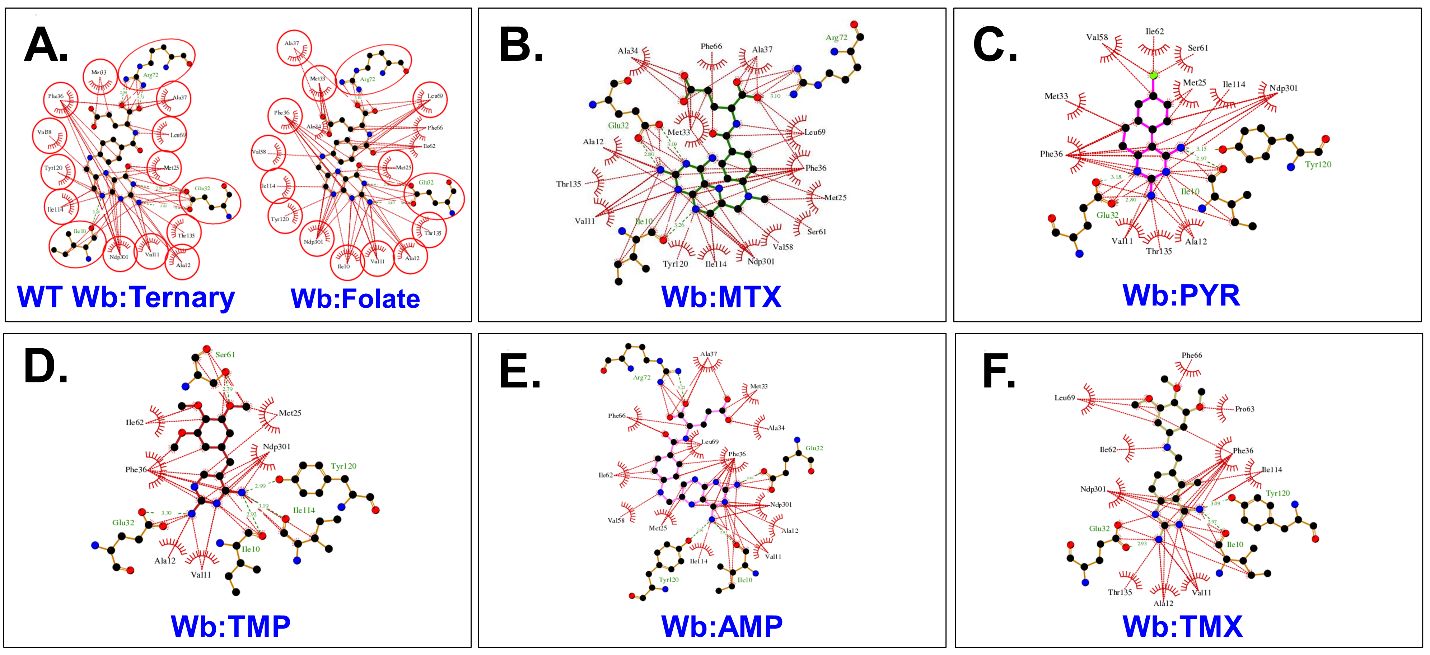
**

**Fig I.** Ligplot images generated for docking models obtained from Autodock Vina.


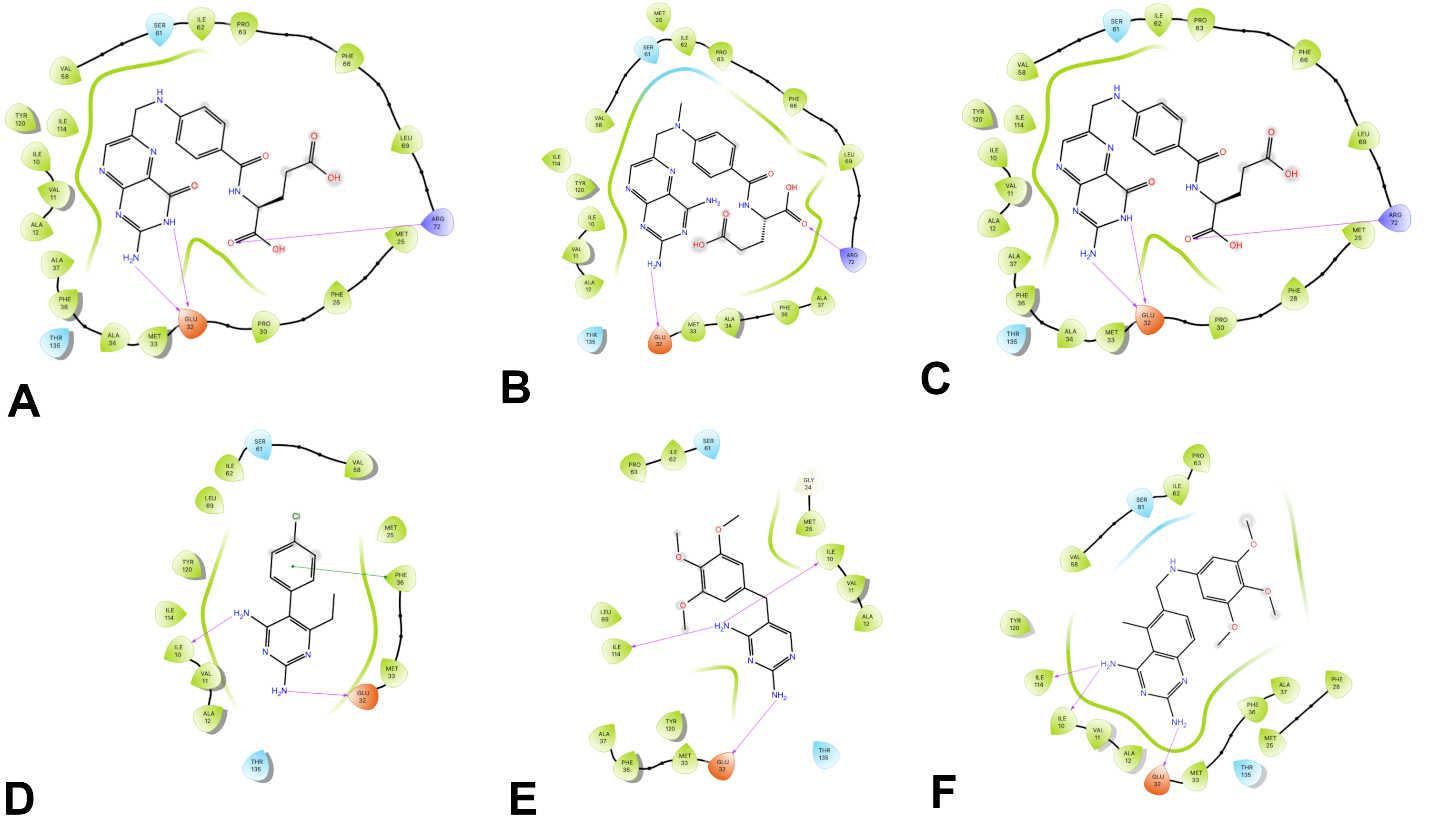


**Fig J.**  Ligand interaction diagrams generated for docking models obtained from Glide. A) *Wb*DHFR-Folic Acid; B) *Wb*DHFR-Methotrexate; C) *Wb*DHFR-Aminopterin; D) *Wb*DHFR-Pyrimethamine; E) *Wb*DHFR-Trimethoprim; F) *Wb*DHFR-Trimetrexate. See Fig. 4 for legend.

**
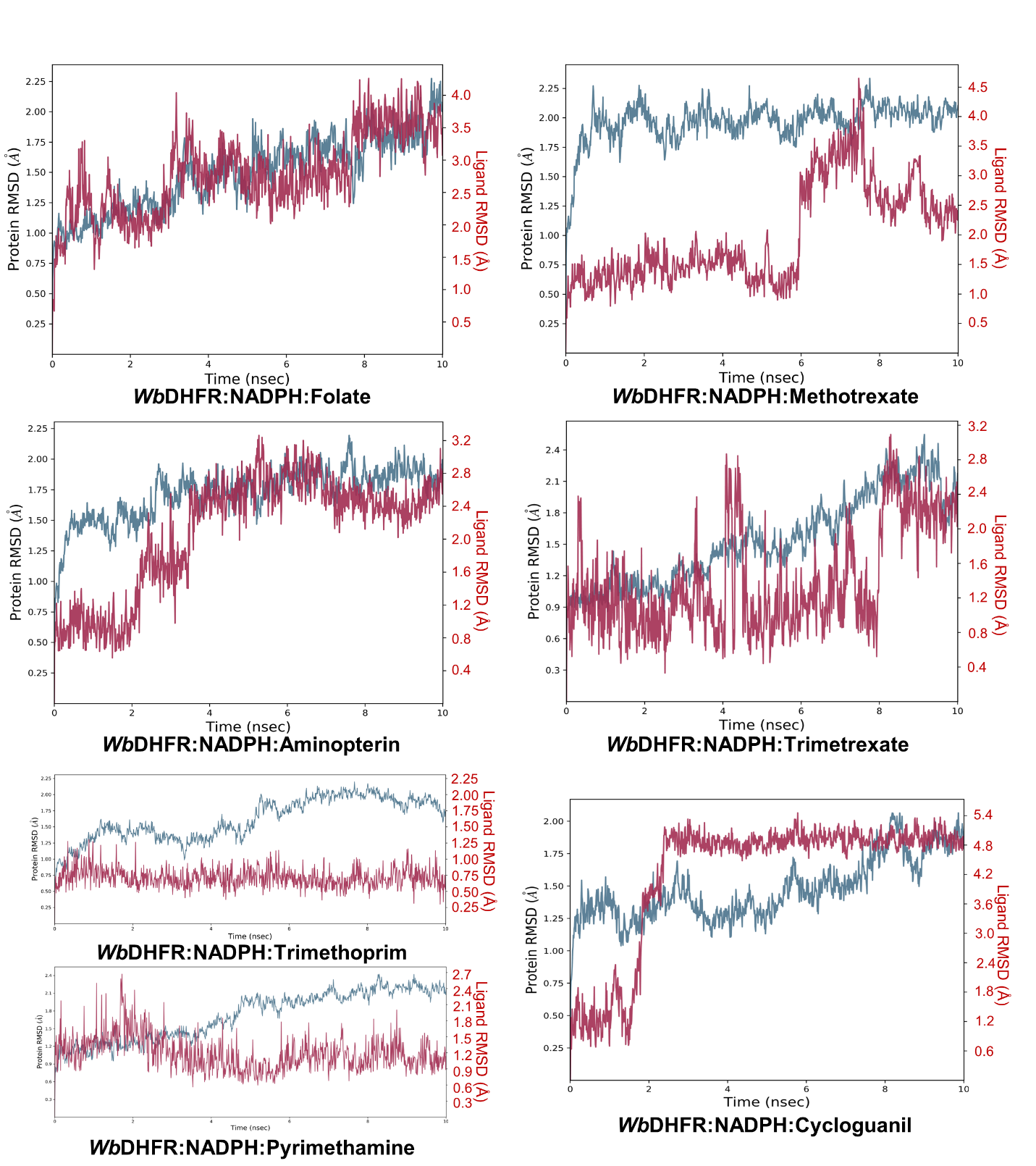
**

**Fig K**: Root mean square deviation (RMSD) values per time in nanoseconds (ns) for all *Wb*DHFR molecular dynamics (MD) simulations conducted at 10 ns. RMSD values for the Cα backbone of *Wb*DHFR (blue) and ligand (red) show equilibration by the end of 10 ns. According to Desmond Simulation Interaction Reports, changes of the order of 1-3 Å for the Cα backbone are acceptable for small, globular proteins such as *Wb*DHFR.


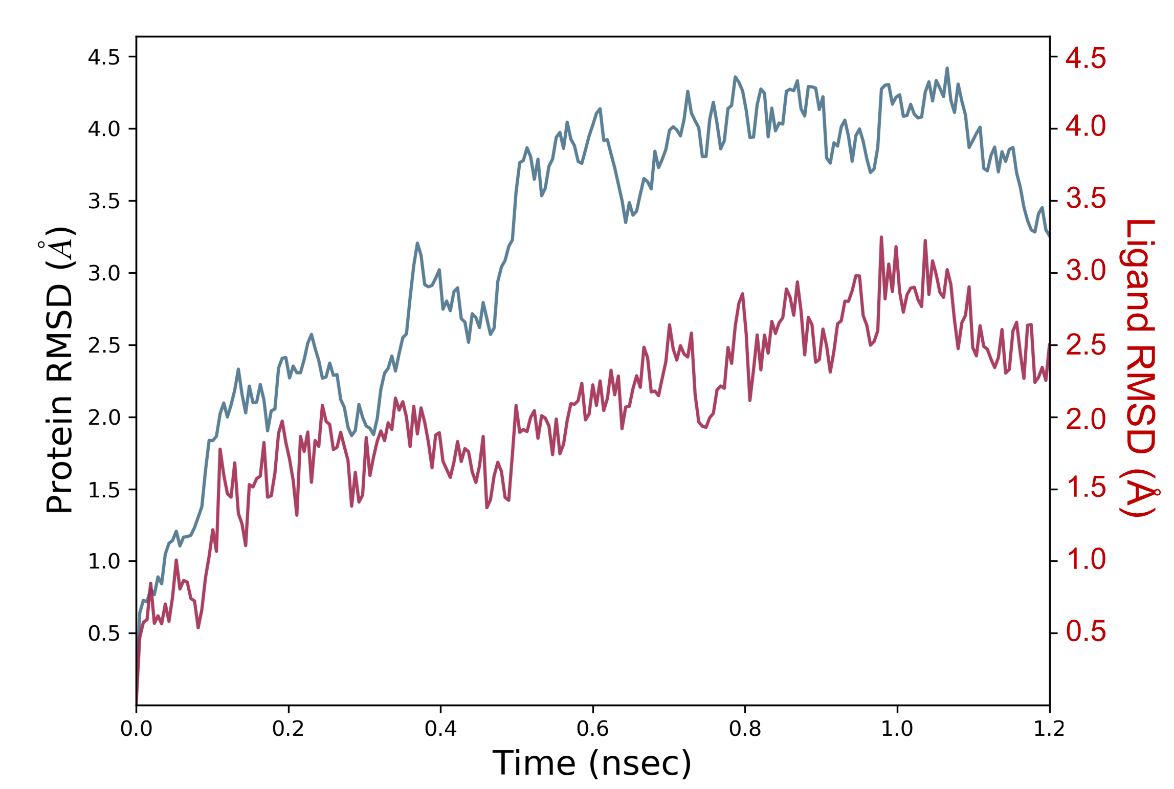


**Fig L.** Root mean square deviation (RMSD) values per time in nanoseconds (ns) for the *Hs*DHFR molecular dynamics (MD) simulations conducted at 10 ns. RMSD values for the Cα backbone of *Wb*DHFR (blue) and ligand (red) show equilibration by the end of 10 ns. According to Desmond Simulation Interaction Reports, changes of the order of 1-3 Å for the Cα backbone are acceptable for small, globular proteins such as *Wb*DHFR.

**Table C.** Comparison of docking scores of antifolates to *Hs*DHFR and *Wb*DHFR using both Glide and Vina.

|  | *Vina score*  *(-kcal/mol)* | | *Glide score*  *(-kcal/mol)* | |
| --- | --- | --- | --- | --- |
| **Ligands** | ***Wb*DHFR** | ***Hs*DHFR** | ***Wb*DHFR** | ***Hs*DHFR** |
| Folate | -9.1 | -9.9 | -9.0 | -10.0 |
| Methotrexate | -8.8 | -9.7 | -9.1 | -9.3 |
| Pyrimethamine | -7.9 | -7.8 | -7.0 | -7.5 |
| Trimethoprim | -7.6 | -8.0 | -6.5 | -7.5 |
| Aminopterin | -8.7 | -10.0 | -8.5 | -9.6 |
| Trimetrexate | -8.6 | -8.7 | -7.9 | -6.5 |

**Method: Determination of IC50 for trimetrexate**

Inhibitor evaluation of trimetrexate on *Wb*DHFR was performed as described previously (Tobias, A. M. et al. *PloS one* 2018, 13 (5), e0197173). Briefly, 100 µM of NADPH, 12.5 nM of recombinant *Wb* DHFR and trimetrexate were added to 1X MTEN buffer at pH 6.0. The assay was initiated by adding DHF to a final concentration of 50 µM and absorbance measurements taken at 340 nm in a SynergyH1 microplate reader. Concentrations of trimetrexate varying from 100 µM down to 1.6 nM were used to generate a dose-response curve where percent enzyme activity was evaluated. The percent activity at each inhibitor concentration was the result of four independent measurements with accompanying standard error shown. The resulting curve was plotted on a log-scale and fitted to a sigmoidal curve using KaleidaGraph software V 4.5.4.

**Fig M.** Evaluation of trimetrexate as an inhibitor of *Wb*DHFR. Enzyme activity plot of *Wb*DHFR against a titration of trimetrexate from 100 µM down to 1.6 nM (see method above). The resulting plot is the average of four independent measurements with error bars representing standard error. The data was fitted to a sigmoidal curve in KaleidaGraph software V 4.5.4 where an IC50 of 491 ± 161 nM was observed. Using the Cheng-Prusoff equation and a Michaelis-Menten constant of 3.7 µM, the KI was calculated to be 33 ± 11 nM.
